# Pathways and therapeutic targets in melanoma

**DOI:** 10.18632/oncotarget.1892

**Published:** 2014-04-08

**Authors:** Emma Shtivelman, Michael A. Davies, Patrick Hwu, James Yang, Michal Lotem, Moshe Oren, Keith T. Flaherty, David E. Fisher

**Affiliations:** ^1^ Cancer Commons, Palo Alto, CA, USA; ^2^ University of Texas MD Anderson Cancer Center, Houston, TX, USA; ^3^ National Cancer Institute, NIH, Washington DC, USA; ^4^ Hadassah Hebrew University Hospital, Jerusalem, Israel; ^5^ The Weizmann Institute of Science, Rehovot, Israel; ^6^ Massachusetts General Hospital Cancer Center, Boston, MA, USA

**Keywords:** melanoma, targeted therapy, immune therapy

## Abstract

This review aims to summarize the current knowledge of molecular pathways and their clinical relevance in melanoma. Metastatic melanoma was a grim diagnosis, but in recent years tremendous advances have been made in treatments. Chemotherapy provided little benefit in these patients, but development of targeted and new immune approaches made radical changes in prognosis. This would not have happened without remarkable advances in understanding the biology of disease and tremendous progress in the genomic (and other “omics”) scale analyses of tumors. The big problems facing the field are no longer focused exclusively on the development of new treatment modalities, though this is a very busy area of clinical research. The focus shifted now to understanding and overcoming resistance to targeted therapies, and understanding the underlying causes of the heterogeneous responses to immune therapy.

## INTRODUCTION

The revolutionary discovery of a striking, if temporary, effect that targeted inhibition of BRAF has on the clinical course of metastatic melanoma has spiked a new wave of research into molecular targets. In addition, it has raised a number of new questions: what are the mechanisms of both inherent and acquired resistance to BRAF inhibitors and the possible ways to overcome this resistance; how is the activating effect of BRAF inhibition on the MAPK pathway in cells with non-mutated BRAF avoided; how should melanoma tumors that have no activating mutations in BRAF, such as tumors with mutated NRAS or NF1, or tumors that are wild type for both BRAF and NRAS, be targeted; which targeted or non-targeted drug combinations should be pursued as determined by the molecular profile of each and every tumor; what is the future of combination targeted therapy and immunotherapy; and many more.

The mutational landscape of melanoma was examined in several large studies employing NGS (next-generation sequencing) and large-scale expression analyses of tumors. The mutation rate of melanoma, on average, exceeds those reported for other aggressive tumors, probably due to the involvement of ultraviolet (UV) radiation in the genesis of superficial cutaneous melanomas. Indeed, the rate of transversions characteristic of UV-induced lesions is much higher in melanoma than the rates of other nucleotide substitutions. The high rate of mutations in melanoma makes it particularly difficult to distinguish between causative (“driver”) mutations and bystander (“passenger”) mutations.

In one recent study [1], a wide range of point-mutation rates was observed: they were lowest in melanomas in which primaries arose on non-UV-exposed hairless skin of the extremities (3 and 14 mutations/megabase [Mb] of genome); intermediate in those originating from hair-bearing skin of the trunk (5-55 mutations/Mb); and highest in a patient with a documented history of chronic sun exposure (111 mutations/Mb). Uveal melanomas, tumors with generally poor prognosis, nevertheless have a fairly low mutation rate [2]. Interestingly, melanomas that are wild type for both BRAF and NRAS have almost five times higher mutational loads than tumors where one of these oncogenes is mutated [3].

The different types of melanoma appear to have distinct sets of relevant somatogenic alterations (Tables 1 and 2). Some of the difference could be attributed to the lack of UV involvement in pathogenesis of acral and mucosal melanomas. Uveal melanoma was recognized as distinct tumor for a long time, and it is reflected in predominance of driver mutations in G protein subunits rather then BRAF or NRAS mutations. Mutational analyses of mucosal melanoma concluded that this subtype shares with cutaneous melanoma relatively few mutations in specific genes, suggesting different mechanisms of carcinogenesis [4]. The rare conjuctival melanoma apparently has a mutational landscape similar to cutaneous melanoma [5], pending confirmation from an analysis of a larger number of tumors.

**Table 1:** Pathways Involved In Melanomagenesis

PATHWAY*	COMPONENTS MUTATED/ACTIVATED	TYPE OF ALTERATION
Receptor tyrosine kinases	KIT	Mutation/amplification
	EGFR	Activation
	MET	Activation; high level of ligand in stroma
	ERBB4	Mutation
	FGFR	Activation; high levels of ligands
Integrin adaptors/ECM signaling	NEDD9/HEF	Amplification
RAS/RAF/MEK/ERK	NRAS	Mutation
	BRAF	Mutation
	MEK1	Mutation
RAS/PI3K/PTEN/AKT/mTOR	PIK3CA	Mutation
	PTEN	Mutation
	AKT1, AKT2	Rare mutation
	AKT3	Amplification
NF1 (PI3K + MAPK pathways)	NF1	Mutation
RHO/RAC/other MAPKs	RAC	Mutation
	MAP3K5 & MAP3K9	Mutation
	PREX	Mutation
Glutamate receptors	GRIN2A	Mutation
	GRM3	Mutation
G proteins other than RAS, effectors of MAPK	GNAQ	Mutation
	GNA11	Mutation
Apoptosis	BCL2A1	Amplification
WNT/β-catenin	CTNNB1	Mutation
CDK	CDK4	Mutation/amplification
	CCND1	Amplification
P53	P14ARF (CDKN2A)	Mutation/deletion
	MDM4	Amplification
RB1	P116INK4A (CDKN2A)	Mutation/deletion
MITF transcriptional program	MITF	Mutation/amplification
MYC transcriptional program	MYC	Amplification/overexpression
ETV1 transcriptional program	ETV1	Amplification
TERT	Promoter region of catalytic subunit	Mutation

* The order of pathways in this Table has no relationship to their significance in melanoma; it is simply from the cell periphery to the nucleus.

**TABLE 2:** Genes Known To Be Altered in Melanoma

PRIMARY SUBTYPES	PATHWAY	ABERRATION	FOUND IN TUMORS WITH…	FREQUENCY	POSSIBLE THERAPIES
**BRAF**	MAPK	Point mutation Gene fusions	NRAS wild type	50-60% rare	BRAFi + MEKi BRAFi + EGFRi + AKTi
**NRAS**	MAPK, PI3K, RALGDS		BRAF wild type	20-25%	MEKi + CDKi
**KIT**	MAPK, PI3K	Point mutation, amplification	NRAS BRAF wild type mostly	1% overall; 10% in in mucosal; 10% in acral	Sunitinib, nilotinib, imatinib
**GNAQ/GNA11**	Gα(q) family of G protein α subunits; MAPK activators	Point mutation	NRAS BRAF wild type	1%; 40-50% each in uveal	MEKi + PI3Ki, enzastaurin
MITF*	Transcription, lineage, cell cycle	Amplification	ALL	20%	HDACi
NF1*	MAPK, PI3K negative regulator of RAS	Mutations, loss of expression	BRAF, NRAS wild type and less often in mutated	4% overall; 25% of BRAF, NRAS wild type	MEKi + mTORi or PI3Ki
TERT*	Telomerase	Mutations in the promoter of catalytic subunit	ND	70-80% overall; 33% primary; 85% metastatic	TERT inhibitors in preclinical
ERBB4	PI3K, MAPK	Point mutation	All types	15-20%	Lapatinib (ERBBi) + PI3Ki
MET	PI3K, MAPK	Activation by stromal HGF	All types	ND	Cabozantinib?
AKT3	PI3K	Amplification	All types	25%	AKTi, PI3Ki, mTORi
PTEN	PI3K	Point mutation or deletions	BRAF mutated; BRAF and NRAS wild type	40-60%	PI3Ki
MAGI	PI3K; stabilizes PTEN	--	All types	--	PI3Ki
TACC	Possibly stimulates PI3K AURKA signaling	--	BRAF and NRAS mutated	5%	PI3Ki, AURKAi
PREX2	RHO/RAC/MAPK; Rac exchange factor	Point mutations	BRAF or NRAS mutated	14%	
RAC1	RHO/RAC/MAPK; Regulator of cell adhesion, invasion, migration	Point mutations	BRAF or NRAS mutated	9% of sun exposed	
MAP2K1, MAP2K2	MAPK (MEK1/2)	Mutations	BRAF mutated; BRAF, NRAF wild type	5%	ERKi
MAP3K5, MAP3K9	RHO/RAC/MAPK	Mutations, loss of heterozygocity	All types	85% and 67%	MEKi, ERKi
MYC	Transcription	Amplification	All types	20-40%	mTORi?
ETV1	Transcription	Amplification	All types	15%	
TP53	Cell cycle, apoptosis	Point mutation	All types	10-20%	
MDM4	Negative regulator of p53	Overexpression	All types	65%	p53-MDM4 i
CDKN2A (P16INK4a, p14ARF)*	Negative regulator of TP53 and RB	Point mutation, deletion	BRAF and NRAS mutated, KIT amplified	30-40%	CDKi
BCL2, BCL2A1	Suppression of apoptosis	Elevated expression, amplification (BCL2A1)	All types	ND 30% (BCL2A1)	BH3 mimetics
CCND1	Cell cycle, G1/S cyclin	Amplifications	More frequent in BRAF, NRAS wild type	11%	CDKi
CDK4*	Cell cycle, G1/S cyclin-dependent kinase	Amplifications	More frequent in BRAF, NRAS wild type	3%	selective CDKi
PPP6C	Catalytic unit of phosphatase, negative regulator of CCND1, Aurora	Point mutations	BRAF and NRAS mutated	12% sun exposed	AURKA CDKi
STK19	Kinase; unknown function	Point mutations	BRAF and NRAS mutated	5 - 10%	
SNX3	Endosome protein sorting	Point mutations	BRAF mutated; BRAF,NRAS wild type	7%	
GRIN2A	Ionotropic glutamate-gated ion channel, NMDA binding	Point mutations	--	25%	
GRM3	Possibly accessory MAPK signaling	Point mutations	ND	13-18%	
TRRAP	Part of histone acetyltransferase complex	Point mutations	ND	10-13%	
ARID2	SWI/SNF Chromatin remodeling, SWI/SNF complex	Inactivating mutations	BRAF, NRAS mutated	7-9%	
BAP1	BRCA1 DNA repair	Inactivating mutations	BRAF, NRAS wild type—uveal	1% overall, 84% uveal	
NEDD9	Integrin adaptor, promotes EMT and migration; metastasis	Amplification	Probably all	50-60%	

Bold font of gene names indicates that these mutations are considered to be driver mutations based on presence in familial melanoma and/or high frequency of mutations is sporadic, even though experimental evidence of their precise role in melanomagenesi is not always available.

* germline mutations in high-risk melanoma families; MC1R mutation is not listed.

Not listed: rare mutations in HRAS, RAF1 and other oncogenes.

Some of the data on the mutation frequency are based on relatively small sample tumor sizes and should be considered with caution. Some data are from the cbioportal.org.

This paper describes genetic alterations that are known to occur in melanoma and information about their role in melanomagenesis, as well as their suitability as targets for therapeutic intervention. The latest information on molecular events underlying inherent or acquired resistance to targeted therapies is also included, along with immunotherapeutic approaches and prognostic methodologies.

Multiple cellular pathways have been implicated in melanomagenesis, ranging from signal transduction to developmental and transcriptional pathways and cell cycle deregulation (Table 1). The search for driver mutations in melanoma continues, with the previously identified subtypes involving BRAF, NRAS, KIT, GNAQ, and GNA11 expanded recently to include NF1 and telomerase (Table 2). Together, these driver mutations are very likely to define the vast majority of molecular subtypes in melanoma and, eventually, the identified mutations in drivers will guide targeted therapy choices. However, for a number of reasons, such as oncogene-induced senescence (discussed below), driver mutations result in a frank tumorigenic phenotype only in the presence of “supporting mutations” that are also listed in Table 2 and described below. It is likely that these supporting mutations have to be targeted along with driver mutations to achieve durable responses. Finally, immunotherapies continue to play an important role in melanoma treatment strategies and are also described here.

### MOLECULAR SUBTYPES OF MELANOMA

The molecular pathways involved in melanomagenesis are summarized in Table 1, and Table 2 lists the known driver and additional mutations/alterations.

### BRAF

BRAF mutations (V600E in 70%-80% of all BRAF mutations in all cancers) occur early in melanomagenesis and are found in a high percentage of melanocytic nevi. Other mutations at the V600 position leading to alternate amino acid substitutions (V600K, V600D, V600R) account for another 5% to 15% of all BRAF mutations. Mutations in BRAF alone do not induce melanoma, and the high frequency of BRAF mutations in benign nevi supports this conclusion [6]. Moreover, expression of the BRAF mutations in preclinical models is associated with the phenomenon known as oncogene-induced senescence (OIS) [7], which results in a cell-cycle arrest in BRAFV600E-expressing cells. Additional genetic events in BRAF-mutant cells, such as deletion of CDKN2A or PTEN, are necessary to elicit a fully cancerous phenotype (reviewed by Flaherty, 2012 [8]).

Overall, about 50% of melanomas of all clinical types have mutations in BRAF. BRAF mutations are more frequent in melanomas that develop in intermittently sun-exposed skin and less so in acral and mucosal melanomas. BRAF mutations are not found in uveal melanomas. Mutations in BRAF and NRAS (~20% of melanomas of the same origin/location as BRAF-mutated melanomas) are almost always mutually exclusive. The V600E mutation in BRAF confers to this kinase the ability to activate MEK (the only known downstream target of BRAF) independent of RAS. A small subset of melanomas that have no mutations in the major driver oncogenes (Table 2) were found to harbor BRAF fusions involving other genes. These might comprise 4 to 8% of the “pan-negative” melanomas, and, while their growth is not affected by BRAF inhibitors, they are sensitive to MEK inhibitors.

The reported results of trials with selective oral BRAF inhibitors showed efficacy in melanoma patients with BRAFV600E mutations [9]. In addition, inhibition of MEK (the only known phosphorylation substrate of BRAF) is considered to be a valid therapeutic intervention for both BRAF- and NRAS-mutated melanoma. However, the success of BRAF inhibitors has not come without problems:

• A significant minority of patients with mutant BRAF show no response to BRAF inhibition [10]. Preclinical results suggest that this group has a variety of additional genomic somatic alterations that confer the resistance phenotype (discussed below).
• Most patients initially responding to BRAF inhibitors develop resistance to those inhibitors within a relatively short period of time (<1 year).
• About 20% of BRAF inhibitor-treated patients developed cutaneous squamous cell carcinomas [10]. These results from a paradoxical activation of MEK-ERK in BRAF wild type cells [11] Photodynamic therapy (PTD) was reported to be effective in treatment of these usually numerous tumors [12]. Recent data suggest that activation of AMPK might attenuate the effects of BRAF inhibition on proliferation of BRAF-wild keratinocytes through inhibitory phosphorylation of BRAF [13]. Accelerated development of RAS-mutant leukemia in a patient treated with vemurafenib was also described [14], further raising concern about the development of other malignancies as a consequence of BRAF inhibition [15].
• Paradoxical effects of BRAFV600 inhibitors in activating wild-type BRAF and/or CRAF. The mechanism of this is thought to be due to conformational changes in wild-type BRAF protein induced by binding of the inhibitors promoting the formation of heterodimers with other RAF isoforms, leading to MEK activation [11]. This effect might trigger growth of new malignant melanoma tumors that have wild-type BRAF, but high expression of RAS-GTP, Akt, and cyclin D1 [16, 17]. A recent study provided a possible mechanism of activation of wild-type BRAF by inhibitors through a relief of inhibitory autophosphorylation [18].

### Treatment approaches to BRAF mutations

The U.S. Food and Drug Administration (FDA) approved the mutant BRAF inhibitor vemurafenib/Zelboraf in 2011. Another inhibitor, dabrafenib/Tafinlar, was approved in May 2013. Dabrafenib showed clinical responses in 59% of patients with V600E/K mutation, including 7% complete responders [19]. A newer inhibitor, LGX818, is in early stages of testing. New inhibitors are in development to eliminate the side effects associated with the paradoxical activation by vemurafenib of the ERK1/2 pathway in wild-type BRAF cells. PLX7904, a new “paradox-breaking” BRAF inhibitor, appears to inhibit ERK1/2 in BRAF-mutant cells without activating BRAF in BRAF wild-type cells [20]. Dosing schedules with BRAF inhibitors were explored in a genetically engineered mouse model (GEMM), where intermittent inhibition of BRAF by vemurafenib works better than continuous treatment [21]. Intermittent dosing of vemurafenib has not yet been tested in clinical trials.

Inhibition of MEK has been explored in clinical trials for melanoma patients with mutant BRAF [22, 23]. A phase III randomized trial comparing MEK inhibitor trametinib to chemotherapy showed a marked improvement in both progression free and overall survival (PFS and OS) in the trametinib arm [23]. Combination of selumetinib/AZD6244 with alkylating agent dacarbazine improved PFS in a randomized clinical trial [24]. MEK inhibitor trametinib showed some clinical responses in BRAF-mutant patients that had undergone previous immune- or chemotherapy, but had no effect in patients who were previously treated with vemurafenib [25]. This strongly indicates that mechanisms involved in development of resistance to mutant BRAF confer resistance to MEK inhibition as well. FDA approved trametinib/Mekinist for melanoma in May 2013.

Most importantly, it is increasingly clear that combination treatments, such as BRAF and MEK inhibition investigated in a recently reported clinical study [26], have potential for a robust and lasting clinical response in the treatment of BRAF-mutant melanoma. Combination dabrafenib+trametinib trial responses were not only more numerous compared to dabrafenib alone, but also more durable. This combination is now being considered for approval by the FDA. BRAF inhibitors are explored in a number of clinical trials in combination with inhibitors of MEK, including four phase III trials (NCT01689519, NCT01597908, NCT01682083, NCT01682083), the first of which involves a new MEK inhibitor GDC-0973 or cobimetinib. Other early-phase combination trials with approved BRAF inhibitors explore addition of inhibitors of PI3K, AKT, CDK, HSP90, RTKs and other targets, as well as immunotherapy.

Targeting ERK1/2, the kinases at the lower end of the MAPK cascade, is an emerging therapeutic strategy, with several candidate drugs in preclinical development. ERK inhibitors have an important advantage of being potentially active in the setting of acquired resistance to inhibitors of BRAF or MEK [27].

### BRAF mutations other than V600

A number of BRAF mutants other than V600 were identified in human cancers [28]. Some of these have a relatively low kinase activity compared to BRAFV600E [29]. Three mutants with low activity—G469E, G466A, and N581S—were shown to still activate ERK via a mechanism involving their ability to strongly activate CRAF[29]. The low-activity BRAF mutations G469E/D594G that signal through CRAF were later identified in a panel of melanoma cell lines [30]. Analysis of BRAF exon 15 in 49 tumors negative for BRAFV600 mutations, as well as other known driver mutations in KIT, NRAS, GNAQ, and GNA11, showed that two (4%) harbored L597 mutations and another two involved BRAF D594 and K601 mutations [30].

Melanomas with BRAF mutations other than V600 have not been specifically targeted in clinical trials. Kinase inhibitor sorafenib is a better inhibitor of CRAF than mutant BRAF; sorafenib induced apoptosis *in vitro* and in a xenograft model *in vivo* in tumors with G469E/D594G [30]. A patient with BRAF(L597S)-mutant metastatic melanoma responded significantly to treatment with the MEK inhibitor TAK-733 [31]. Another patient with this mutation responded to trametinib in the phase I clinical trials mentioned above [22].

### NRAS

Approximately 20% of melanomas have mutations in the GTPase NRAS. NRAS and BRAF mutations are almost always mutually exclusive. Therapeutic approaches targeting mutant NRAS directly have not been successful. Combination treatments targeting the downstream effectors of NRAS remain a viable option.

### Potential treatment approaches to NRAS mutations

The pathways downstream of NRAS that could be targeted simultaneously in NRAS-mutant melanoma include, but are not limited to, MEK, PI3K/mTOR, and cell-cycle-related targets. PTEN abnormalities are rarely found in NRAS-mutant tumors [32]. Monotherapy with the MEK inhibitor MEK162 showed limited partial responses (20%) in NRAS-mutant patients and represents the most active single-agent targeted therapy evaluated to date [33]. A recent study identified the basis of different activity of MEK inhibitors in BRAF versus KRAS mutant cancers. Unlike trametinib-like inhibitors that inhibit phosphorylated MEK and are effective in the setting of BRAFV600 mutants, the new class of inhibitors, like GDC-0623, inhibit feedback activation of MEK by RAF, and are therefore more efficacious in the setting of mutant KRAS [34]. It is likely that GDC-0623, which is currently in a phase I clinical trial, might be efficacious in melanomas with mutant NRAS.

Preclinical studies indicate several potential points of intervention

• NRAS-driven melanoma in genetically engineered mice responded only to the combination of MEK and PI3K/mTOR dual inhibitors out of 16 treatment combinations tested [35]. Combined targeting of MEK and PI3K was superior to MEK and mTOR inhibition in NRAS-mutant melanoma cell lines and xenografts [36]. A number of clinical trials examining this combination are ongoing.
• In an inducible model of NRAS-mutant melanoma, genetic ablation of NRAS triggered cell-cycle arrest and apoptosis, while pharmacological inhibition of MEK activated apoptosis, but not cell-cycle arrest. CDK4 was implicated as a key driver of these differences and combined pharmacological inhibition of MEK and CDK4 *in vivo* led to substantial synergy in therapeutic efficacy in a mouse model [37]. The phase I/II trial NCT01781572 with MEK inhibitor MEK162 and CDK inhibitor LEE011 for NRAS-mutant melanoma is ongoing.
• Sensitivity of NRAS-mutant cell lines to MEK inhibitors was shown to be associated with expression of AHR (aryl hydrocarbon receptor) *in vitro* [38].
• A study of combinatorial drug interactions *in vitro* pinpointed the combination of simvastatin with a CDK inhibitor as the only fairly effective cytotoxic treatment for NRAS-mutated melanoma cell lines [39].

The combinations of inhibitors to target NRAS-activated signaling through MEK and PI3K, MEK and AKT, MEK and PI3K/mTOR, as well as MEK and VEGF-receptor inhibition, are now in early phase clinical trials. Only a few trials specifically target melanomas with NRAS mutations, but a number of trials use combinations of agents or single agents that could have therapeutic benefits in this subgroup of melanoma. Single agents in phase I or early phase II trials include inhibitors of CDK (PD0332991, dinaciclib, LY2835219, BAY1000394, LEE011), the Notch pathway (RO4929097), and Aurora kinase A (MLN8237/alisertib, GSK1070916A) (Supplemental Table 2).

### GNAQ and GNA11

Activating mutations in GNAQ and GNA11, encoding members of the Gα(q) family of G protein α subunits, are driver oncogenes in uveal melanoma [40, 41]. Mutations in GNAQ and GNA11 are mutually exclusive and are present in the vast majority of uveal melanomas [42]. GNA11 has a stronger association with metastatic uveal melanoma than GNAQ. Mutations in these GTP-binding proteins activate the MAPK pathway.

### Potential treatment approaches to GNAQ and GNA11 mutations

A randomized phase II clinical trial compared the MEK inhibitor selumetinib (AZD6244) with temozolomide (NCT01143402) with results showing superiority of selumetinib in terms of PFS and overall response rate (ORR), but not OS (J Clin Oncol 31, 2013 suppl; abstr CRA9003). GNAQ mutation promotes resistance *in vitro* to selumetinib, but the combination of selumetinib with the ATP-competitive mTOR inhibitor AZD8055 might be more promising [43]. A trial of the MTOR inhibitor everolimus and the somatostatin-receptor-activating peptide pasireotide/SOM232 is recruiting patients (NCT01252251).

Inhibition of both PI3K and MAPK, but neither of them singly, might also work in uveal melanoma as seen from *in vitro* experiments [44]. No trials involving this combination are ongoing.

Enzastaurin and AEB071, PKC inhibitors, have shown some activity against uveal melanoma cell lines *in vitro* [45, 46]. PKC is involved in signal transduction from GNAQ to MEK. AEB071 is tested in a phase I clinical study NCT01430416 as a single agent, and will be examined in combination with trametinib in NCT01801358.

Recently, it was shown that the guanine nucleotide exchange factor Trio is involved in mitogenic signaling through GNAQ and GNA11. Trio is essential for activating Rho- and Rac-regulated signaling pathways acting on JNK and p38, thereby transducing proliferative signals from Gα(q) to the nucleus independently of phospholipase C-β [47]. These findings might open new avenues for treatment of uveal melanoma.

### MITF

Microphthalmia-associated transcription factor MITF is a lineage survival oncogene, amplified in 20% of melanoma cases [48]; amplification of MITF is associated with a reduced 5-year survival. MITF is mutated in some familial melanomas [49]. The variant MITF(E318K) co-segregates with affected individuals in familial melanoma and is likely to be a gain-of-function (GOF) mutation. It abolishes a SUMO-ilation site on MITF that reportedly acts to inhibit transcriptional activity of MITF. Transcriptional targets of MITF have been identified and, besides a large number of lineage-specific transcripts, include genes related to regulation of cell cycle, among them CDK2 [50]. Considering that MITF itself is currently not a druggable target, inhibition of CDK2 is a plausible aim in melanoma with MITF aberrations.

Other candidate targets in the MITF program include receptor tyrosine kinase TYRO3, which regulates expression of MITF in a SOX-10-dependent manner [51]. Ubiquitin-specific protease 13 (USP13) was shown to be responsible for MITF deubiquitination and stabilization. USP13 is essential for melanoma growth *in vitro* and *in vivo* and might be another target in MITF-mutated melanoma [52].

Hypoxia-inducible factor HIF1α was also reported to be a transcriptional target of MITF[53], and its expression is apparently stimulated by MITF with the well-known consequences of stimulating tumor survival, angiogenesis, and metastases. Paradoxically, expression of MITF itself is reduced under hypoxic conditions in normal melanocytes and melanoma via direct binding of transcription repressor DEC1, which is activated by HIF1α [54]. This suggests a negative feedback loop. A recent study implicated HIF1 factors in promoting melanoma invasion and metastases without affecting proliferation of the primary tumors [55].

MITF is regulated by the transcription factor ATF2. Primary melanoma specimens that exhibit a high nuclear ATF2:MITF ratio were found to be associated with metastatic disease and poor prognosis [56]. ATF2 control of melanoma development is mediated, in part, through its negative regulation of SOX10 and consequently, of MITF transcription. In human patients, virtually all congenital nevi and melanomas are SOX10 positive. SOX10 silencing in human melanoma cells suppresses neural crest stem cell properties, counteracts proliferation and cell survival, and completely abolishes *in vivo* tumor formation [57].

SOX10, PAX3, and MITF participate in regulation of MET (HGF receptor), which is expressed at high levels in human melanoma [58]. MITF and PAX3 bind directly to the MET promoter; coexpression of these three proteins is found in melanoma biopsies [59].

PPAR-γ coactivators PGC-1α and PGC-1β are critical components of the melanogenic system governed by MITF. Melanomas with high expression of PGC-1α exhibit increased expression of mitochondrial respiration complexes and increased oxidative phosphorylation [60]. The high MITF-high PGC-1α expressing cells have an increased capacity to withstand oxidative stress, and, unlike PGC-1α low cells, respond poorly to ROS-inducing agents such as PEITC or piperlongumine [60]. In addition, polymorphism studies reveal expression quantitative trait loci (eQTLs) in the PGC-1β gene that correlate with protection from melanoma in humans [61].

BCL2A1, a lineage-specific anti-apoptotic protein in the BCL2 family, is amplified and overexpressed in MITF-high melanomas, and confers resistance to BRAF inhibitor [62]. Suppression of BCL2A1 might have clinical benefit in this group of melanomas [62].

A recent paper proposed targeting of tyrosinase (MITF transcriptional target) by first inducing expression of MITF in melanoma cells using the well-known anti-folate drug methotrexate. The second step is to target the now high-MITF expressing cells with a designed pro-drug that is converted to a cytotoxic anti-folate drug by the MITF-induced tyrosinase [63]. The death of thus targeted cells should then ensue as a consequence of dTTP depletion. There are many questions regarding the rationale and execution of this strategy, including how methotrexate induces MITF expression, how to reconcile the known oncogenic role of MITF in melanoma to further increase its expression, and how targeting DHFR—a long explored target in cancer—could overcome the acquired resistance long associated with methotrexate. The loss of tyrosinase expression in advanced amelanotic tumors would need to be considered as well.

The crystal structure of MITF was resolved recently, paving the way for the future targeting of MITF in melanoma and other cancers [64].

### Potential treatment approaches to MITF amplification

These findings on MITF-mediated pathways remain in the domain of preclinical research, although targeting MET receptor is feasible because MET inhibitors (cabozantinib et al.) are in clinical trials for cancers other than melanoma. For now, panobinostat/ LBH589 (HDAC inhibitor) is in early clinical testing in melanoma (NCT01065467), and also involves examining the effect of panobinostat on MITF expression. The rationale behind investigating HAD inhibitors is supported by a study demonstrating efficacy of the HDAC inhibitor in suppressing MITF expression *in vitro* and *in vivo* [65].

A new study opened a possibility of targeting the nuclear translocation of ATF2 by inhibiting PKCε, which phosphorylates ATF2 and induces its transport to the nucleus. The two compounds found to promote cytoplasmic localization of ATF2 were identified in an image-based screen [66].

### KIT

KIT is a receptor tyrosine kinase activated by binding of the cytokine stem cell factor (SCF); mutations and amplification of KIT are found in particular subsets of melanoma. KIT mutations activate signal-transduction pathways (MAPK and PI3K) that ultimately lead to cell proliferation. Mutations in KIT are found in mucosal, acral, and chronically sun-exposed skin melanomas [67]. KIT mutations are rare in melanoma, but the availability of selective inhibitors for KIT has prompted interest in targeting this oncogene. These inhibitors—imatinib, sunitinib, nilotinib, and dasatanib—were developed for different cancers (chronic myeloid leukemia, gastrointestinal stromal tumors) and different kinases, but they showed activity against KIT. Approximately 70% of KIT mutations identified in melanoma are found in exon 11, most commonly L576P, and in the kinase domain in exon 13, most often K642E [68]. The majority of mutations of KIT found in melanoma also occur in other cancers that are responsive to KIT inhibitors.

### Treatment approaches to KIT mutations

Targeting KIT with imatinib has demonstrated remarkable efficacy in patients with gastrointestinal stromal tumors (GIST), but initial trials in melanoma were unsuccessful, clearly due to the absence of selection of patients with aberrations in KIT [69-71]. Dasatinib was tested as a single agent in unselected melanoma patients and also showed poor response and high toxicity [72]. Nevertheless, case reports continued to surface that demonstrated the efficacy of imatinib in melanoma patients with specific KIT genetic aberrations, such as mutation K642E [73] and a small duplication [74]. Two patients whose melanomas harbored L576P responded to dasatinib [75]. Even when alterations in KIT are present in tumors, not all are predictive of responses to kinase inhibitors. Overexpression of KIT in melanoma was found not to confer sensitivity to imatinib [76]. Sunitinib malate was tested in another trial; again, no correlation was observed between the degree of KIT expression and longer PFS or OS [77].

Recently, trials of imatinib and nilotinib have shown promising results in trials where patients were selected for KIT mutations. Phase II trials of imatinib in melanoma produced only modest responses to treatment, but 9 of the 10 partial responses (PRs) were observed in patients with mutations in exons 11 or 13 [78]. Another phase II trial of imatinib in 28 patients with alterations in KIT produced durable responses in 16% of patients, predominantly in those with KIT mutations versus those with amplifications [79]. Sunitinib induced responses in 3 out of 4 patients with KIT mutations in exons 11 and 13, but only in one out of six patients with KIT amplification [80]. Preliminary results of a trial with nilotinib were published when it showed durable responses in patients with KIT mutations [81]. A recent phase II trial of imatinib in selected patient population with mucosal, acral, or chronically sun-damaged melanoma concluded with an overall disease-control rate of 50%, but the responses were observed in 77% of patients with mutated KIT and only in 18% of those with amplified KIT. The best ORR was, respectively, 54% versus 0% [82]. Preexisting NRAS mutations conferred intrinsic resistance to imatinib [82] and to sunitinib (Minor et al., 2012) in patients with KIT mutation. It is becoming clear that inhibitors of KIT should be used in selected patient populations whose tumors have KIT mutations in exon 11 and 13 but not where KIT is not mutated but amplified [83].

At least 10 ongoing trials test the activity of the aforementioned inhibitors in KIT-mutated melanoma as single agents. Planned and ongoing trials include combinations of KIT inhibitors with other targeted drugs and immunotherapy. Trial NCT01738139 recruits patients for the combination of imatinib with ipilimumab (an immunomodulatory antibody to CTLA4) in patients with various tumors that have somatic alterations of KIT.

### NF1/neurofibromin

NF1 is a tumor suppressor regulating signaling from RAS; germline mutations in NF1 deregulate both PI3K and MAPK pathways and result in familial neurofibromatosis. Some neurofibromatosis patients with inactivation of NF1 develop melanomas. Moreover, NF1 expression is low in 47% of uveal melanomas [84] and allelic losses are seen in other types of melanoma [85]. Mutations in NF1 are enriched in melanoma that have wild-type BRAF and NRAS (25% of those had NF1 mutations as reported by Hodis [32], albeit a relatively small number of patients was analyzed), suggesting that they could be considered as driver mutations in this subset that was, until now, not amenable to targeted therapy approaches. In addition, NF1 mutations or suppression occur in human melanomas that harbor concurrent BRAF mutations [32, 86]. Mutations in the NF1 cooperate with BRAF mutations in a mouse model of melanomagenesis by suppressing BRAF-induced senescence (OIS), promoting melanocyte hyperproliferation and enhancing melanoma development. Knockdown of NF1 *in vitro* promotes activation of both KRAS and CRAF.

### Potential treatment approaches to NFI mutations

In recent studies NF1 could be successfully targeted in mouse models with a combination of MEK and PI3K/mTOR inhibitors [86]; a combination of irreversible RAF inhibition and MEK inhibition was also effective *in vivo* [87].

### Telomerase

Two very recent studies—one based on analysis of genomic alterations in a melanoma-prone family, the other based on analysis of the genomic sequence data from melanoma tumors—have revealed new, frequent, and unexpected mutations in the regulatory regions of the catalytic subunit of telomerase [88, 89]. Mutations were found in 33% primary and 85% metastatic tumors in the first study versus 71% of all tumors in the second study. Mutations create a new binding motif for TCF/ETS transcription factors and result in an increased transcription from TERT promoter. Mutations in the promoter region of TERT are considered to be driver mutations because of their association with familial melanoma and high frequency in sporadic melanoma. TERT promoter mutations are not limited to melanoma, and were found in 16% of tumor cell lines from diverse cancers [89].

### Potential treatment options to telomerase mutations

Telomerase inhibitor imetelstat sodium/GRN163L (antisense oligonucleotide) is trialed in breast cancer and a telomerase vaccine GV1001 in nonsmall cell lung cancer (NSCLC).

### Receptor tyrosine kinases and PI3K pathway

Activation of the PI3K pathway serves to overcome OIS that is associated with mutant BRAF. PTEN aberrations are often associated with the presence of BRAFV600E, and cooperate with mutant BRAF in a GEMM [90], most likely by overcoming OIS associated with mutant BRAF. In support of this notion, while BRAF mutations are present in both nevi and melanoma sections of contiguous nevi-melanoma biopsies, activation of PI3K (through loss of PTEN expression or activation of AKT3) was detected in the melanoma portions only [91]. This indicates that the AKT3/PI3K pathway is activated during progression to malignant melanoma, most likely as a means of overcoming OIS. PI3K pathway activation serves as a rate-limiting event and dual inhibition of PI3K/mutant BRAF eliminated cells resistant to BRAF inhibition *in vitro* [91]. Several mutations in different components of the PI3K pathway, from receptor tyrosine kinases (RTK) to PTEN and to AKT3, have been described.

### ERBB4

Mutations in this tyrosine kinase receptor are found in 19% of melanomas, based on the results of targeted sequencing of the tyrosine kinase family in seven melanoma tumors [92]. The pattern of mutations, however, did not exhibit recurrent hot spots. Subsequent studies have reported either much lower incidence (2% of 271 tumors) of ERBB4 mutations in melanoma [93], or lack of any in sampling of 117 tumors [94]. Nevertheless, lapatinib, a reversible inhibitor of EGFR, ERBB2, and other kinases is currently in a clinical trial for advanced melanoma with ERBB4 mutations (NCT01264081).

### MET

Mutations in MET have not been described in melanoma, but there is strong evidence that this RTK is involved in melanoma growth and metastases (see below: WNT pathway and HGF expression in stroma). Copy number gains involving MET locus in melanomas were documented [95]. SU11274, an inhibitor of MET, has shown significant activity in a xenograft model [96]. Cabozantinib, an inhibitor of MET and other RTKs is in clinical trials for a variety of cancers with deregulated RTK signaling. In melanoma, cabozantinib will be trialed in combination with vemurafenib (NCT01835184).

### PDGFRα

Mutations in this RTK were detected in 4.6% of Chinese melanoma patients (in 351 biopsies sequenced in the study), mostly in acral and mucosal tumors. All 18 mutations identified were different, which casts a doubt on their functional significance. However, five of the 12 analyzed had a gain of function effect on PFGFRa *in vitro*. These were more sensitive to crenolanib than to imatinib [97].

### MERTK

MER, a receptor tyrosine kinase (RTK) sharing a family relationship with TYRO3 and AXL, was found to be expressed at increasing levels during progression from nevi to metastatic melanoma. Stimulation of MER with its ligand GAS6 leads to activation of MAPK, PI3K, and JAK/STAT pathways. Inhibition of MERTK with a synthetic compound UNC1062 inhibits invasion and induces apoptosis in melanoma cells *in vitro* [98]. MERTK and AXL are expressed alternatively in melanoma [99]. MERTK-expressing melanoma cells are more proliferative than AXL-expressing cells, though the latter are more invasive [99].

### AKT

Deregulated Akt3 activity was shown to promote development of malignant melanoma; amplifications of Akt3 were detected in melanoma [100]. Alterations of AKT1 and AKT2 are rare, but genetic gain of Akt3 is seen in 25% of melanoma tumors. A screen of 137 melanomas and 65 cell lines identified an activating mutation E17A in AKT1 (one patient) and AKT3 (one patient and two cell lines), all with concurrent BRAF mutations [101]. High activity of AKT3 was shown to promote progression of BRAFV600E-positive nevi to melanoma [102]. A trial combining MK2206 (Akt inhibitor) in combination with AZD6244 (the MEK inhibitor selumetinib) is in progress (NCT01519427).

### PTEN

PTEN is a well-known tumor suppressor affected in approximately 25% to 30% of melanoma, most commonly via allelic loss and focal deletions [32, 103]. PTEN is also deregulated in melanoma via loss of ZEB2, a competitive endogenous RNA (ceRNA) [104]. In addition, disruptions of MAGI2, a protein that associates with and stabilizes PTEN, occur in melanoma [1]. PTEN aberrations are often associated with the presence of BRAFV600E, and cooperate with mutant BRAF in a GEMM [90], most likely providing an OIS inhibitory function by activating the PI3K pathway. In this model, growth of melanoma could be inhibited by combined treatment with PD325901 (MEK inhibitor) and rapamycin (mTOR inhibitor). PTEN-inactivating aberrations are not associated with NRAS mutations, perhaps because the latter lead to activation of PI3K in the absence of this pathway's mutations. PTEN mutations and copy losses are associated with lower PFS in response to inhibition of mutant BRAF in patients [105]. PTEN (or rather lack of PTEN) was shown to play a key role in the predisposition to melanoma in individuals carrying certain variants of melanocortin-1 receptor (MC1R). The mechanisms through which MC1R variants associated with red hair and fair skin predispose to melanoma were not understood until recently, when it was shown that wild-type but not variant MC1R protein associates with PTEN and protects it from degradation after UVB exposure. Disrupted MC1R-PTEN association was shown to be responsible for the oncogenic transformation promoted by MC1R variants in presence of BRAFV600E [106].

### Other PI3K pathway mutations and alterations

Mutations in other PI3K pathways genes MTOR, IRS4, PIK3R1, PIK3R4, and PIK3R5 were detected in 17% of BRAFV600 and in 9% of NRAS-mutant tumors [107], even though their functional significance remains to be determined. Phosphoinositide-dependent kinase-1 (PDK1) is a serine/threonine protein kinase that phosphorylates and activates kinases of the AGC family, including AKT. Increased expression of PDK1 was observed in a large cohort of melanoma samples compared to nevi, and deletion of PDK1in a GEMM of melanoma BRAFV600E/PTEN-/- significantly delayed development of tumors and metastases [108].

### MAPK pathway downstream of BRAF

### MEK1 and MEK2

Sequencing of seven melanoma cell lines and donor-matched germline cells found MAP2K1 and MAP2K2 (MEK1 and MEK2, respectively) mutations, resulting in constitutive ERK phosphorylation and higher resistance to MEK inhibitors. Screening of a larger cohort of melanoma tumors revealed the presence of recurring somatic MAP2K1 and MAP2K2 mutations at an overall frequency of 8% [109].

### IQGAP1: a scaffold protein in MAPK pathway as a possible target

There are new data indicating that it might be possible to target the MAPK pathway without direct inhibition of enzymatic activity of kinases in this pathway. It is well known that kinases usually depend on scaffold proteins that assemble signaling complexes. One of these scaffold proteins, IQGAP1 is essential for activity of ERK1/2, and disruption of IQGAP interaction with ERK using a small peptide inhibited RAS- or BRAF-driven tumorigenesis and even overcame resistance to vemurafenib [110].

### RAC pathway

RAC1 is a Rho GTPase, a GTP exchange protein known to affect the cell cytoskeleton and motility. Its role in conveying oncogenic signaling from mutant NRAS in melanoma was described (Li et al. [111] and references therein). Recently P29S mutations in the conserved switch domain were described in melanoma, in 5% of tumors in the experimental set [32, 112], and their functional role *in vitro* was confirmed. Mutations were also found in other Rho family members-RAC2 (P29L), RHOT1 (P30L), and in CDC42 (G12D) -making RAC pathway a target of frequent hotspot mutation. Activity of RAC pathway is affected not only by mutations, but is regulated by available cellular pool of GTP, which is controlled by several enzymes including GMPR (guanosine monophosphate reductase) which ultimately depletes cellular GTP pools. High levels of GMPR downregulates Rho-GTPase levels, and expression of GMPR is lost in invasive melanoma where activity of RHO pathway contributes to invasiveness of [113].

### MAP3K5 and MAP3K9

These MAP3 kinases are directly downstream of Rac and Rho signaling activated by various stress signals. Mutations and the loss of heterozygosity of MAP3K5 and MAP3K9 in 85% and 67% of melanoma samples, respectively, suggest inactivation of these kinases. Indeed, mutants MAP3K5 I780F and MAP3K9 W333* variants had reduced kinase activity *in vitro*. Overexpression of these mutants reduced the phosphorylation of downstream MAP kinases, while siRNA-mediated depletion of MAP3K9 in melanoma cells led to increased cell viability after temozolomide treatment, suggesting that decreased MAP3K activity acts as a pro-survival adaptation [114].

### PREX2

Mutations of phosphatidylinositol-3,4,5-trisphosphate-dependent Rac exchange factor 2 were found in 14% of a cohort of 107 tumors [1]. Functional studies *in vitro* have confirmed a role for PREX2 in melanomagenesis. Presence of mutations in three functional components of RAC pathways (Rho family proteins, MAP3K5 and MAP3K9, PREX2) strongly indicates involvement of RAC pathway in pathogenesis of melanoma.

### Cell cycle and apoptosis-related genes

CDK4, a cell cycle G1/S kinase, and cyclin D1 (CCND1), were shown to be amplified in melanoma [115]. CDK4 mutations are associated with familial melanoma [116]. Significantly, CDK4 pathway is deregulated in most melanomas as a consequence of increased activity of ERK or deletion of CDK4 inhibitor p16INK4A (below). In a clinical study of FOLFIRI (chemotherapeutic agent) with the nonselective CDK inhibitor flavopirodol in solid tumors, one melanoma patient had a complete response [117]. Targeted CDK inhibitors are in clinical development (PD0332991 (palbociclib), SCH 727965 (dinaciclib), LY2835219, BAY1000394, LEE011), and are currently in clinical trials for various advanced cancers including melanoma.

### TP53 pathway

TP53 mutations are found in 19% of melanoma tumors [32], a frequency relatively low compared to other cancers, raising the possibility that melanomas uses alternative ways to overcome p53-mediated tumor suppression. Indeed, several alterations in genes affecting p53 activity have been discovered in melanoma. Among them are the long known mutations in p14ARF[118], overexpression of MDM2 [119], amplification of MDM4 in melanoma[120], elevated expression and the anti-apoptotic role of p53-related protein p63 [121], and increased expression of iASPP [122] -all of which act to inhibit the function of p53. This means that the rare “p53 mutant subtype” could now be expanded to a “p53 pathway aberrations” subtype.

CDKN2A locus is frequently deleted in melanoma of all primary subtypes [123-125]. Two tumor suppressors are encoded within this locus: p14ARF, which activates p53 through inhibition of its major negative regulator MDM2; and p16INK4a, a cyclin-dependent kinase inhibitor that activates retinoblastoma (RB) through negative regulation of CDK4. Loss of CDKN2A was reported to occur in 16% to 41% of sporadic melanoma and with a high frequency in familial melanoma [118]. Loss of CDKN2A and CCND1 correlate with poor responses to dabrafenib [105].

### MDM2

This protein binds to p53 and promotes its degradation and inactivation. MDM2 is considered to be the major negative regulator of p53. Recently, it has been reported that in a fraction of melanoma tumors MDM2 expression is upregulated owing to the silencing of a microRNA, miR-18b, that targets Mdm2 mRNA [126]. The authors suggest that targeted overexpression of miR-18b could be considered as a novel strategy for activation of the p53 pathway in melanoma.

### MDM4

In a recent study it was shown that MDM4, a negative regulator of p53, is upregulated in about 65% of melanomas, and that melanocyte-specific MDM4 overexpression enhanced tumorigenesis in a mouse model of melanoma induced by NRAS [120]. Inhibition of the MDM4-p53 interaction restored p53 function in melanoma cells, resulting in increased sensitivity to cytotoxic chemotherapy and to inhibitors of the BRAFV600E mutation. MDM4 could be a key determinant of impaired p53 function in human melanoma and a promising target for anti-melanoma combination therapy [120].

Potential treatment of melanoma harboring inactivated wild-type p53 would involve inhibition of CDK4, and MDM2-p53 and MDM4-p53 interactions. The MDM2-p53 interaction has attracted serious efforts to develop specific inhibitors, of which Nutlin analogues are the more advanced. The new generations of Nutlin-based drugs are in clinical studies for various solid tumors. Encouraging results were reported for one of them, RG7112, in early clinical testing for liposarcoma and other tumors [127]. Nutlin3, an early prototype inhibitor of p53-MDM2 interaction, was shown to synergize with vemurafenib in inducing cell death in melanoma cell lines and inhibiting tumor growth *in vivo* [128]. An alternative approach under development is based on the use of stapled peptides against MDM2/MDM4, which exhibit high potency and selectivity [129]. A potent inhibitor of MDM2 and MDMX interactions, ATSP-7041, reactivated TP53 function and inhibited tumor growth *in vitro* and *in vivo* [130].

P63, a protein related to p53, has been reported to have an anti-apoptotic role in melanoma, which is mediated through its interaction with p53. P63 is expressed at high levels in melanoma cell lines and clinical samples and prevents translocation of p53 to the nucleus [121]. This study further expands the role of the aberrations of the p53 pathway in melanomagenesis.

iASPP is a conserved ankyrin repeat protein that shuttles between nuclear and cytoplasmic compartments, and nuclear iASPP is found in proliferating cells. The nuclear iASPP was shown to inhibit the pro-apoptotic function of p53 [122]. High levels of nuclear iASPP were observed in metastatic melanoma versus primary melanoma [131]. The researchers found that iASPP is phosphorylated by cyclin B/CDK1, which promotes its nuclear localization and binding to p53, inhibiting its pro-apoptotic function. Prevention of iASPP phosphorylation by CDK1 inhibitor or knock-down of iASPP induced apoptotic death in melanoma cell lines, which is further enhanced by Nutlin3 (inhibitor of p53-MDM2 interaction and degradation of p53). Furthermore, inhibition of BRAF with vemurafenib, or MEK with UO126, potentiated effects of Nutlin3 and cyclin B /CDK1 inhibition, inducing apoptosis of melanoma *in vitro* and *in vivo* [131]. These findings indicate an alternative strategy for combining therapies that target MAPK pathway and cyclin B/CDK1 in wild-type p53 melanoma.

### NFkB

As in many, many other tumors, the NFkB pathway is activated in melanoma, but systemic inhibition of NFkB might have catastrophic general adverse effects and toxicity. A new facet of NFkB activation was described recently in drug-treated melanoma cells that might acquire a senescent secretory phenotype; the latter results in a pro-inflammatory and pro-metastatic phenotype characterized by production of CCL-2. Activated NFkB and PARP-1 contribute to this phenomenon and could be involved in the therapeutic failure [132].

### BCL2

There is abundant literature that documents elevated BCL2 expression in melanoma and its contribution to melanoma and melanocyte cell survival. A member of this family, BCL2A1, was shown to be amplified in 30% of melanoma and contribute to resistance to BRAF inhibition [62] (see section “Intrinsic Resistance to BRAF and MEK Inhibitors”). Another member of BCL2 family, BCL2L12 is mutated in 4% of melanomas, but mutation is synonymous. mRNA from this mutated BCL2L12 is stable due to failure of targeting by specific miRNA and leads to higher levels of protein. BCL2L12 binds and inhibits tumor suppressor TP53 [133].

Attempts to target BCL2 with antisense RNA in melanoma patients have not been successful. Obatoclax, a drug inhibiting interaction of BCL2 with pro-apoptotic proteins Bax and Bak, is in clinical trials for other malignancies. BH3 mimetics to inhibit function of anti-apoptotic proteins, such as BCL2 and BCL-xL, are still subjects of significant interest. One of them, ABT-737, resensitized both melanoma cell lines *in vitro* and tumors in the *in vivo* model to common chemotherapeutics (including the only FDA-approved chemotherapeutic for melanoma, dacarbazine), leading to marked BIM (Bcl-2-interacting mediator of cell death) -mediated apoptosis. ABT-737 may be a beneficial adjuvant therapy to improve melanoma response rates when conventional chemotherapy is the only option [134].

### β-catenin and WNT pathway

A number of reports have heavily implicated WNT signaling in melanoma progression and metastases. β-catenin fortifies the cadherin-based adhesion at the plasma membrane, but, when detached from cadherins, activates transcription of target genes, frequently with oncogenic consequences. Rare mutations in β-catenin and in other members of the WNT signaling family were identified in malignant melanoma 10 years ago [135]. β-catenin suppresses expression of p16INK and cooperates with NRAS in transformation to a frank melanoma [136]. WNT5a, in particular, by binding to the Frizzled4- LRP6 complex, activates ARF6 (guanosine triphosphatase adenosine diphosphate ribosylation factor 6), leading to displacement of β-catenin from N-cadherin in melanoma. This stimulates signaling from β-catenin and increases invasiveness [137]. Non-canonical WNT5A signaling was implicated in the plasticity of melanoma cells expressed as phenotype switching from expression of ROR1 to ROR2 during hypoxia and acquisition of invasive characteristics [138]. Moreover, the increased WNT5a signaling and expression of ROR2 are associated with metastases and increased resistance to BRAF inhibition [138].

In a mouse melanoma model based on PTEN loss and BRAFV600E mutation, β-catenin was shown to be a central mediator of metastases as well as a regulator of both MAPK and PI3K pathways. Recent findings established WNT signaling as a metastasis regulator in melanoma [139]. Mutant BRAF signaling is thought to inhibit WNT/β-catenin signaling. Endogenousβ-catenin was apparently required for the efficacy of PLX4720 *in vitro*; activation of WNT/β-catenin signaling was found to enhance the anticancer activity of PLX4720 *in vitro* and *in vivo* [140].

Negative regulation of WNT/β-catenin signaling by MAPK pathway was confirmed in an additional study [141]. Treatment of BRAF-mutant and NRAS-mutant melanoma lines with WNT3A and the MEK inhibitor AZD6244 induces apoptosis. The susceptibility of BRAF- and NRAS-mutant lines to apoptosis correlated with negative regulation of Wnt/β-catenin signaling by ERK/MAPK signaling and dynamic decreases in abundance of the downstream scaffolding protein, AXIN1 [141]. WNT inhibitors such as PRI-724 (inhibitor of interaction between β-catenin and CBP) and OMP-54F28 (a fusion protein antagonistic to Fzd8) are starting to enter clinical testing in tumors other than melanoma.

### Transcriptional factors in melanoma

### MYC

This universal oncogene and transcriptional master regulator is overexpressed or present at increased copy numbers in 41% or more of melanoma tumors [95]. It is currently considered not to be druggable.

### ETV1

This transcription factor from the ETS family was implicated as an oncogene in melanoma and copy-gain numbers were found in 40% of cases examined, with amplification of ETV1 in 13% to18% of cases [142].

### Other significant genetic abnormalities in melanoma

NEDD9, an integrin adaptor protein related to P130CAS, and a member of a family implicated in pathogenesis of a variety of cancers, was identified as a bona fide melanoma metastasis gene in melanoma. NEDD9 enhanced invasion *in vitro* and metastasis *in vivo* of both normal and transformed melanocytes, and was frequently overexpressed in metastatic melanoma relative to primary melanoma [143]. Fifty-seven percent of melanomas were found to have amplification of NEDD9 [95].

PPP6C is a serine-threonine phosphatase, mutated in 12% of sun-exposed melanomas exclusively with BRAF or NRAS mutations [32, 112]. PPP6C is the catalytic unit of a phosphatase complex that negatively regulates activity of the mitotic Aurora kinase, a known oncogene. Most mutations map in the conserved domain that is involved in interaction with the regulatory subunit of the complex.

TACC1 (transforming acidic coiled-coil containing protein 1) is mutated in 5% of an experimental set of 121. TACC1 is known to stimulate the PI3K and RAS pathways and interact with Aurora kinase, which is notable considering that PPP6C mutations (above) also inactivate Aurora kinase [32]. At least 16 Aurora kinase inhibitors are in clinical studies, of which two (MLN8237/alisertib, GSK1070916A) are investigated in melanoma.

BAP1 (BRCA1-associated protein-1/ubiquitin carboxy-terminal hydrolase) is involved in metastatic progression of ocular and cutaneous melanoma. BAP1 is a known tumor-suppressor gene. BAP1 mutations are frequently found in uveal melanoma [144]. Germline BAP1 mutations have recently been associated with an increased risk of several cancers, including atypical melanocytic tumors [145] and uveal melanoma [146]. Uveal melanoma might be sensitive to HDAC inhibitors [147], and a clinical trial of HDAC inhibitor vorinostat/SAHA in uveal melanoma is ongoing (phase II NCT01587352).

**SF3B1**. Codon 625 of the SF3B1 gene, encoding splicing factor 3B subunit 1, is consistently mutated in low-grade uveal melanomas with good prognosis [148]. Mutations of SF3B1 are associated with disomy 3 [149], are mutually exclusive with BAP1 mutations and lead to aberrant splicing of transcripts of a number of genes [2].

SNX31 was identified as one of the 11 new genes mutated in melanoma [32]. It encodes a poorly characterized sorting nexin 31 protein. It could be a Ras effector protein that selectively binds GTP-loaded H-RAS [150].

### STK19

A 5% mutation rate of this kinase gene with unknown functions is seen in melanoma [32].

### LKB1/STK11

LKB1 might be a central kinase that integrates energy metabolism and tumor growth, in part through activation of the family of AMPK kinases. Germline mutations in LKB1 (STK11) are associated with the Peutz-Jeghers syndrome (PJS), which includes aberrant mucocutaneous pigmentation, and somatic LKB1 mutations occur in 10% of cutaneous melanoma [151]. Somatic inactivation of LKB1 with K-Ras activation in murine melanocytes led to highly metastatic melanoma with 100% penetrance. AMPK was shown to attenuated BRAF activity through direct phosphorylation and disruption of its functionally important association with a scaffold protein KSR [13]. Downstream events of LKB1 inactivation, in addition to AMPK-related effects, included increased phosphorylation of the SRC family kinase YES, increased expression of WNT target genes, and expansion of a CD24(+) cell population in melanoma with increased metastatic behavior *in vitro* and *in vivo* [152]. Dasatinib, an SRC inhibitor, was shown previously to exhibit a higher activity towards YES rather than SRC, and could be a promising treatment for LKB1-mutated melanoma [152]. Metformin, an indirect activator of AMPK, the downstream target of LKB1, is currently in a clinical trial in combination with vemurafenib (NCT01638676). Phenformin, another antidiabetic drug that is no longer in use, also an AMPK activator, has a synergistic activity with vemurafenib in BRAF mutant melanoma *in vitro* and *in vivo* [153]. It should be noted that earlier studies found that metformin could be accelerating growth of NRAS mutant cell lines, and that vemurafenib could have antagonistic effect in some BRAF mutant melanoma cell lines, in particular those that are resistant to vemurafenib [154, 155]

ARID2 is a component of the SWI/SNF chromatin-remodeling complex. Loss-of-function mutations were found in 7% of melanomas. Targeted search identified mutations in other members of the ARID family (ARID1B, ARID1A, SMARCA4), altogether amounting to 13% of the experimental set [32].

TRRAP2. Identified mutations occur in 4% of the melanoma set examined. TRRAP functions as part of a multiprotein coactivator complex possessing histone acetyltranferase activity that is central to the transcriptional activity of p53, c-Myc, and E2F1 [156].

GRIN2A encodes the glutamate N-methyl-D-aspartic acid (NMDA) receptor subunit ε-1 that is part of the class of ionotropic glutamate receptors and bears the agonist binding site for glutamate. GRIN2A was found to be mutated in 25% of melanomas [156]; this was confirmed in another study [114]. Many mutations are missense or nonsense; therefore, it is unlikely to behave as a canonical oncogene.

GRM3 is a metabotropic glutamate receptor, a G-protein-coupled receptor (GPCR) that activates phospholipase C upon ligand binding. It was found to be mutated in melanoma through exome-capture analysis of GPCR genes [157]. Mutated GRM3 was shown to contribute to the proliferation and invasiveness of melanoma cells *in vitro* and induce an increased phosphorylation of MEK. AZD-6244, an inhibitor of MEK, was able to reduce cell proliferation by inducing apoptosis *in vitro*. There is some interest in using available inhibitors of glutamate release for treatment of melanoma, since one of them, riluzole, was shown to inhibit growth of melanoma cells *in vitro* and *in vivo* [158]. Even though riluzole was shown to inhibit growth of cell lines expressing GRM1, a clinical trial is ongoing to explore the antitumor activity of riluzole in melanoma without prior analysis of GRM3 status.

Phosphoglycerate dehydrogenase PHGDH serves to divert glycolytic carbon into serine and glycine metabolism in some cancer cells to supply the increased biosynthetic needs of transformed phenotype [159]. The same study found that PHGDH is recurrently amplified in a genomic region of a focal copy number gain most commonly found in melanoma. PHGDH catalyzes the first step in the biosynthesis of serine and subsequent generation of nucleotides. Melanoma cell lines with amplified PHGDH had increased flux through the serine pathway. This pathway, as well as proliferation of cells with high PHGDH, was sensitive to short hairpin RNA (shRNA)-mediated knockdown of PHGDH [159].

WEE1. Cell cycle regulatory kinase Wee1 is upregulated in melanoma and is associated with poor prognosis [160]. Selective inhibitor of Wee1 MK-1775 showed somewhat promising results as a single agent in previously treated patients with metastatic melanoma, but additional trials are not being conducted at this time.

NUAK2 (AMPK-related kinase). High levels of expression of NUAK2 were found in patients with acral melanoma and are associated with increased risk of relapse [161]. NUAK2 knockdown suppresses melanoma cell growth *in vitro* and tumorigenicity *in vivo* and has been proposed as a new oncogene in acral melanoma.

### Various noncoding RNAs and other epigenetic alterations

A number of reports found significant roles for miRNA, ncRNA, or ceRNA in pathogenesis of melanoma; for example, ZEB2, a ceRNA for PTEN, upregulates expression of this tumor suppressor in melanoma. Abrogated ZEB2 cooperates with BRAFV600E to promote melanomagenesis [104]. ADAR1, a protein of the family known as adenosine deaminase acting on RNA, is substantially downregulated during metastatic progression of melanoma. ADAR1 was found to regulate expression of numerous miRNAs, as well as of the key miRNA processing protein DICER. Two miRNAS were implicated in silencing of ADAR1 itself [162]. These findings reaffirm the significant contribution of epigenetic miRNA regulation to melanoma pathogenesis.

Strategies to inhibit or increase expression of ncRNAs in clinical setting are only beginning to emerge. A growing interest in epigenetic alterations such as chromatin remodeling, DNA methylation, and histone modification regarding their role in melanomagenesis, might lead to the identification of novel therapeutic targets (reviewed by van den Hurk[163]).

### Intrinsic resistance to BRAF and MEK inhibitors

Resistance to BRAF inhibitors could be intrinsic (as in lack of response to selective BRAF inhibitors in patients with BRAF-mutated tumors) or acquired (development of resistance after treatment with BRAF or MEK inhibitors). Both are of the utmost concern. The current understanding of the origins of intrinsic resistance, as well as possible approaches to overcoming it, are addressed here (see Table 3).

**Table 3:** Intrinsic Resistance to BRAF and MEK Inhibitors

MOLECULAR CHANGE	CONFIRMED IN PATIENT BIOPSIES?	DRUGS TO OVERCOME RESISTANCE
Resistance to Vemurafenib		
Loss of PTEN and consequent loss of BIM expression [165],[91], [105]	Yes	PI3Ki + MEKi
NF1 mutation or loss of expression[86], [87]	Yes	MEKi/mTORi
Loss of CDKN2A and amplification of CCND1[168], [105]	Yes	CDKi
Metabolic signature [171]	Yes, high FDG uptake is associated with response to BRAFi	
MET and SRC signaling [169]	Observed in patient-derived lines	METi?
Production of HGF by stroma [173]	Yes	METi?
Expression of antiapoptotic proteins, BCL2 and BCL2A1 [175] [62]	Yes	BH3 mimetics
Elevated expression of MITF and PGC1a [172]	Yes	OXPHOSi
Elevated expression of FOXD3 and ERBB3 [176], [177]	No	ERBB2/3i
Expression of Hsp90 [179]	No	XL888, Hsp90i
Persistent activity of mTORC1 [181], [182]	Yes	mTORi
Resistance to MEK Inhibitors		
Signaling through TGF-β/SMURF2/PAX3 and MITF [183]	No	
Downregulation of PTEN, activation of PI3K [184]	No	PI3Ki + MEKi

It is well established that BRAF mutations play a role in melanomagenesis; however, without additional genetic alterations, tumor development is restricted by OIS. As described in the earlier section, and in Table 1, additional genetic alterations are present in BRAF-mutant tumors, some of which serve to overcome OIS, and could play a role in inherent resistance to mutant BRAF and MEK inhibitors. These two groups overlap, as could be expected. The necessity of targeting multiple signaling pathways to overcome drug resistance of aggressive melanoma was demonstrated *in vitro* [164]. Genomic analyses and the informed choice of combinatorial approaches analyzed in preclinical models are critical in selecting the right combination of targeted therapies in a personalized approach to melanoma treatment. In general, in addition to the MAPK pathway that is deregulated in most melanomas, the other targets might include any of the ones listed in Table 1. Experimental evidence indicating involvement of several pathways/genes in inherent resistance to BRAF inhibition is cited below in Table 3.

### PI3K/AKT

Mutations of the PI3K pathway are frequent in the BRAF-mutant setting. These have been shown to overcome BRAFV600E-induced OIS and contribute to inherent resistance to BRAF inhibitors [91]. In particular, loss of PTEN and consequent loss of expression of the pro-apoptotic BIM that is distantly regulated by PTEN were implicated in inherent resistance to BRAF inhibitor *in vitro* [165] and to dabrafenib in patients [105]. PTEN-negative or AKT3-overexpressing melanomas do not undergo apoptosis in response to BRAF inhibition and do not upregulate pro-apoptotic protein BIM. PLX4720 was found to stimulate AKT signaling in the PTEN-, but not the PTEN+, cell lines. A clinical trial with inhibitors of both MAPK and PI3K showed promise in patients with various solid tumors [166].

A recent study demonstrated an essential role for ERK-phosphorylated MEK1 (pT292) in membrane recruitment of PTEN and consequent negative regulation of AKT [167]. Inhibition of BRAFV600-MEK1-ERK therefore might lead to the inhibition of the restraining role that this pathway has on activity of PI3K/AKT pathway via PTEN.

### CDK4 pathway

Increased cyclin D expression mediates inherent resistance to mutant BRAF inhibition [168], and copy number changes in CDKN2A, CCND1 correlated with the shortened duration of PFS in patients treated with dabrafenib [105].

### NF1 mutations

In a mouse model, NF1 ablation decreases the sensitivity of NF1 wild-type melanoma cell lines to BRAF inhibitors, and NF1 is lost in tumors from patients following treatment with these agents. Nf1/BRAF-mutant tumors are resistant to BRAF inhibitors, but are sensitive to combined MEK/mTOR inhibition [86]. In another study, NF1 mutations were documented in BRAF-mutant tumor cells that were intrinsically resistant to BRAF inhibition, and in melanoma tumors from patients exhibiting resistance to vemurafenib, thus demonstrating the clinical significance for NF1-driven resistance to RAF/MEK-targeted therapies [87].

### MET and SRC

The activation of MET and SRC signaling was detected in two patient-derived melanoma cell lines with BRAFV600E that were resistant to BRAF inhibitor PLX4032. MET or SRC, respectively, were targeted with siRNA or drugs in combination with PLX4032. This was effective in inhibiting cell growth and reducing cell invasion and migration, indicating a functional role for MET and SRC signaling in primary resistance to PLX4032 [169].

### Metabolic signature of melanoma sensitive to mutant BRAF inhibition

An interesting study could not find correlation between sensitivity to PLX4032 and genetic profiles in a panel of BRAF-mutant cell lines. However, the sensitive cell lines had a more profound inhibition of FDG uptake upon exposure to PLX4032 than resistant cell lines. This indicates that melanomas with a higher dependence on glycolysis might be more sensitive to mutant BRAF inhibition. This also indicates that FDG-PET could be useful in assessing sensitivity to BRAF inhibitors [170]. Indeed, a clinical study that incorporated FDG-PET monitoring of responses to vemurafenib strongly indicated that there is a positive correlation between responses to therapy (PFS) and reduction in the uptake of FDG [171].

### MITF-PGC1a axis in resistance to BRAF inhibition

Inhibition of BRAFV600 and, to a lesser extent of MEK, were found to induce expression of genes involved in citric acid cycle and oxidative phosphorylation (OXPHOS) in melanoma. Search for factors regulating OXPHOS revealed that PGC1a is upregulated in resistant melanoma lines [172]. PGC1a, in turn, was found to be a direct transcriptional target of MITF [60, 172]. MITF expression and, as a consequence, PGC1a levels, are upregulated in melanoma lines and in tumors of patients treated with Vemurafenib. Inhibition of BRAF leads to inhibition of glycolytic pathway for ATP production, but MITF-expressing melanomas can undergo a bioenergetics adaptation via MITF-PGC1a-OXPHOS upregulation [172]. The authors suggest targeting OXPHOS in this group of patients prior to use of MAPK inhibitors because lines selected to resistance to vemurafenib have elevated levels of PGC1a.

### Tumor stroma influence

An important study demonstrated production of HGF by stromal cells in patients with melanoma, which resulted in activation of the HGF receptor MET, reactivation of the MAPK and PI3K pathways, and resistance to BRAF [173]. Addition of growth factors was shown to rescue various tumor cells lines from killing by kinase inhibitors including vemurafenib in melanoma [174]. High production of HGF was observed in the stroma samples from patients who had a poor response to the inhibition of mutant BRAF. In a cellular model, co-treatment with BRAF and HGF or MET inhibitors reversed drug resistance [173].

### BCL-2

Inhibition of anti-apoptotic protein Bcl-2 might have a potential role in the future studies aimed to prevent the development of resistance to BRAF inhibition. The BH3 mimetic ABT-737 (inhibiting both Bcl2 and Bcl-xL) sensitizes human melanoma cells to apoptosis induced by selective BRAF inhibitors, but does not reverse acquired resistance *in vitro* [175].

### BCL2A1

Amplification of the family member, BCL2A1 in 30% of melanoma, was shown to contribute to resistance to BRAF inhibition [62]. BCL2A1 expression is apparently restricted to melanocytic lineage as it is indirectly controlled by MITF, and because of this its expression is limited to high-MITF-expressing melanomas. Obatoclax, inhibitor of the BCL family, helps to overcome the resistance of cell lines with amplified BCL2A1 to BRAF inhibition.

### FOXD3-ERBB3

Transcription factor FOXD3 was shown to be upregulated when mutant BRAF is inhibited in melanoma cell lines [176]. Subsequent work revealed that FOXD3 directly activates expression of ERBB3, which contributes to resistance to vemurafenib via activation of PI3K pathway, with involvement of ERBB2. The latter finding indicates a possibility of targeting ERBB2 alongside BRAF to overcome resistance [177].

Chaperone Hsp90 is required for the stability of several of the oncoproteins that mediate RAF inhibitor resistance. Inhibitors of Hsp90 may be effective in patients with intrinsic or acquired resistance to BRAF inhibition [178]. In lab studies, treatment of melanoma cells with XL888, the inhibitor of Hsp90, induced apoptosis more effectively than dual MEK/PI3K inhibition in several different models of resistance [179]. Multiple proteins, including PDGFRβ, COT, IGFR1, CRAF, ARAF, S6, cyclin D1, and AKT, were degraded as a result of inhibition of Hsp90, which led to the nuclear accumulation of FOXO3a, an increase in BIM expression, and the downregulation of Mcl-1. XL888 was effective against NRAS mutant melanoma cells *in vitro* and in xenografts, most likely by decreasing protein levels of AKT, CDK4, and WEE1 [180]. XL888 is now in clinical studies in combination with vemurafenib (NCT01657591). Another non-geldanamycin Hsp90 inhibitor, STA-9090 (ganetispib), is also in clinical trials.

### mTORC1

In BRAF-mutant melanoma sustained mTORC1 signaling could be driven by alternative mechanisms of ERK activation or concomitant activation of the PI3K-Akt pathway, thus promoting resistance to RAF and MEK inhibitors. Activity of mTORC1 after treatment of melanoma cells *in vitro* with BRAF or MEK inhibitors was found to be a faithful predictor of the response as evidenced by the phosphorylation status of ribosomal protein s6 [181]. . In patients, suppression of phospho-S6 was significantly associated with improved PFS [182]. Quantitation of p-S6 could serve as a biomarker to guide treatment of BRAF mutant melanoma. It also suggests that simultaneous inhibition of mutant BRAF and mTORC1 might be of clinical value. Repeated biopsies would be needed to predict the effect of MAPK inhibition on s6 phosphorylation and treatment responses. Report presented at a recent meeting described the feasibility of using fine needle tumor aspirates for evaluation of the s6 phosphorylation status (Corcoran R, AACR-NCI-EORTC International Conference on Molecular Targets and Cancer Therapeutics, Abstract C137). Several early-phase clinical trials examining MEK inhibitors with PI3K/mTOR inhibitors are recruiting patients (phase I NCT01363232, NCT01337765, NCT01390818, NCT01392521).

### Intrinsic resistance to MEK inhibitors

### Signaling through TGF-β/SMURF2/PAX3 and MITF

In an *in vitro* study, cells sensitive to MEK inhibition demonstrated increased transforming growth factor β (TGF-β) signaling. Melanoma cells resistant to the cytotoxic effects of MEK inhibitors counteracted TGF-β signaling through overexpression of the E3 ubiquitin ligase SMURF2, which resulted in increased expression of the transcription factors PAX3 and MITF. High MITF expression protected melanoma cells against MEK inhibitor cytotoxicity. The study also found increased SMURF2 expression in advanced stages of melanoma [183].

Activation of the PI3K pathway was seen as a factor in resistance of a panel of melanoma cell lines to novel MEK inhibitor E6201. The sensitivity of cell lines to MEK inhibition correlated with wild-type PTEN and mutant BRAF [184].

### Acquired resistance to BRAF and MEK inhibitors

Mechanistic studies have provided insights into the development of resistance through two major mechanisms: new mutations in the RAF-MAPK pathway itself and changes in other oncogenic pathways, mainly RAS and PI3K, that relieve melanoma cells from reliance on BRAF signaling [185]. Emergence of resistance in different tumors from the same patient could involve different mechanisms and genes [186].

The identification of mechanisms of acquired resistance to BRAF inhibitors usually involves selection of surviving cells/clones from BRAF-mutant cell lines *in vitro* after treatment with a BRAF inhibitor and identification of newly acquired mutations or other somatic changes. Biopsies from relapsed patients are then analyzed for the presence of these *in vitro*–identified changes. Alternatively, biopsies are subjected to massive sequencing for the identification of newly acquired mutations/aberrations. A clinical study that examined mechanisms of developing resistance to vemurafenib concluded that it resulted mainly from reactivation of MAPK signaling [187]. Based on findings described below, it appears that many of the confirmed mechanisms of resistance are indeed in this category, though activation of pathways “parallel” to BRAF-MAPK has been documented (Table 4).

**Table 4:** Acquired Resistance to BRAF and MEK Inhibitors

MOLECULAR CHANGE	CONFIRMED IN PATIENT BIOPSIES?	DRUGS TO ADD TO OVERCOME RESISTANCE	PATHWAYS ACTIVATED
Resistance to vemurafenib			
Expression of splicing variant of BRAF lacking exons 4-8 [189] [186]	Yes, in 6/19 relapsed patients		MAPK
Amplification of BRAF [188]	Yes, in 4/20 relapsed patients		MAPK
Activation of EGFR/SFK/STAT3 [190]	Yes	EGFRi, SRKi	RTK
Activation of FGFR3-RAS pathway [191]	in vitro only	FGFRi	RTK
Activation of IGFR1-PIK3 pathway [192]	Yes	IGFRi	RTK
Activation of ERBB3 – AKT [194], [177]	in vitro only	HER3i, HER2i	RTK
Activation of PDGFRβ [193]	Yes	RTKi	RTK
Loss or mutations in NF1 [86, 87]	Yes	MEKi + PI3Ki + RAFi	RAS
Mutation of NRAS [193] [186]	Yes	MEKi?	RAS
Activation of MAP3K8/COT, ERK activating kinase independent of MEK [199]	Yes		MAPK
MEK1 mutations [200] [187, 208]	Yes		MAPK
AKT3 [195]	in vitro only	AKTi	PI3K
AKT1 Q79K [196]		MAPKii+PI3Ki	PI3K-AKT
Activation of ERK independently of MEK through PI3K [197]	in vitro only	PI3Ki	PI3K
Loss of expression of RND3 [201]	in vitro only		RHO
High levels of insulin receptor substrate 1 (IRS1) [202]	in vitro: established and patient-derived cell lines	New IRS inhibitors in preclinical	RTK
Activation of cAMP dependent melanocyte lineage program [349]	in vitro; CREB upregulation in biopsies	MAPKi+HDACi	Melanocyte lineage program
Resistance to MEK inhibitors			
MEK1 mutation 187	Yes	ERKi	MAPK
Activation of PI3K/AKT	in vitro only	IGFRi, AKTi, mTORi	PI3K
Resistance to BRAF and MEK inhibitors			
Mutations in MEK2 and amplification of BRAF [210],[211]	Yes	BRAFi + MEKi +mTORi; ERKi	MAPK

### New aberrations in BRAF

Unlike the experiences with other molecularly targeted therapies of oncogenic kinases, treatment with BRAFV600E inhibitors did not lead to the emergence of gatekeeper mutations in BRAF itself. However, amplification of mutant BRAF was detected in 4 of 20 patients who developed resistance to vemurafenib [188].

Analysis of a subset of cells resistant to vemurafenib (PLX4032) *in vitro* detected the 61-kDa variant form of BRAFV600E, p61BRAFV600E, which lacks exons 4 to 8, a region that encompasses the RAS-binding domain. p61BRAFV600E shows enhanced dimerization in cells with low levels of RAS activation, as compared to full-length BRAFV600E that acts as a monomer. The p61BRAFV600E-splicing variants lacking the RAS-binding domain were identified in the tumors of 6 of 19 patients with acquired resistance to vemurafenib. These data determined a novel mechanism of acquired resistance in patients: expression of splicing isoforms of BRAFV600E that dimerize in a RAS-independent manner [189].

### Activation of signaling through RTKs

### EGFR/SFK/STAT3

BRAF inhibitor–mediated activation of EGFR/SRC family kinase/STAT3 signaling was shown to mediate resistance in BRAF-mutant melanoma cell lines and was confirmed in patient biopsies. *In vitro* treatments with an EGFR inhibitor in combination with a BRAF inhibitor, or monotherapy with dasatinib, appeared to overcome this resistance and could deliver therapeutic efficacy in drug-resistant BRAF-mutant melanoma patients [190].

### FGFR3-RAS

Upregulation of FGFR3 signaling in selected vemurafenib-resistant cells was described *in vitro*. Signaling through FGFR3 activated the MAPK pathway through RAS, but these cells were still sensitive to inhibition of MEK or pan-RAF inhibition [191].

### IGF-1R/PI3K pathway

Mutations in the IGF-1R/PI3K pathway were identified as another way for developing resistance to the BRAF inhibitor SB590885. IGF-1R/PI3K signaling was enhanced in resistant melanomas; combined treatment with IGF-1R/PI3K and MEK inhibitors induced death of BRAF inhibitor-resistant cells. Increased IGF-1R and pAKT levels in a post-relapse human tumor sample were consistent with the proposed role for IGF-1R/PI3K-dependent resistance to BRAF inhibitors [192].

Activation of PDGFRβ signaling was shown to occur during selection for BRAF inhibitor resistance *in vitro*. The clones with PDGFRβ activation were also resistant to MEK inhibition, implying that activation of PDGFR bypasses the tumor dependence on RAF signaling entirely [193]. These findings were validated in resistant tumors [193].

### ERBB3 signaling

Enhanced ERBB3 signaling promoted resistance to RAF pathway inhibitors in cultured melanoma cell lines and in mouse xenograft models [177]. ERBB3 was transcriptionally activated by FOXD3, and increased ERBB3 signaling was dependent on ERBB2. Targeting ERBB2 with lapatinib in combination with the BRAF inhibitor PLX4720 reduced tumor burden and extended latency of tumor regrowth *in vivo* versus PLX4720 alone. ERBB3 was shown to be the main RTK undergoing rapid hyperphosphorylation upon either treatment with a BRAF inhibitor or with a MEK inhibitor in another publication [194]. The mechanism of ERBB3 was traced to an autocrine loop that resulted in production of high levels of neuregulin by melanoma cells in response to MAPK pathway inhibition.

### PDGFR β activation

Induction of PDGFRβ RNA, protein, and tyrosine phosphorylation were detected in a cell line model of resistance to vemurafenib as well as in short-term cultures and patient-derived biopsies [193].

NF1 expression is lost in tumors from patients following treatment with BRAF and MEK inhibitors [86]. NF1 mutations were observed in melanoma tumors obtained from patients exhibiting resistance to vemurafenib, as already mentioned. However, cells lacking NF1 retained sensitivity to the irreversible RAF inhibitor AZ628 and an ERK inhibitor [87].

### NRAS

Secondary mutations in NRAS were shown to confer resistance to PLX4032 *in vitro* [186, 193]. These acquired changes were mutually exclusive with PDGFRβ activation observed in the same study. In contrast to PDGFR-activated resistant cells, NRAS-mutated cells were sensitive to MEK inhibitors. Mutations of NRAS were also found in 4 out of 19 patients that developed resistance to PLX4032 [193], and in additional patients in a clinical trial exploring mechanism of acquired resistance to vemurafenib [187]. New mutant BRAF inhibitors, such as PLX7904, have been explored *in vitro*. PLX7904 inhibits ERK1/2 activation in cells with mutant BRAF, but not in cells with wild-type BRAF [20]. Promisingly, PLX 7904 inhibited ERK1/2 phosphorylation in mutant BRAF melanoma cells with acquired resistance to vemurafenib/PLX4720 that is mediated by a secondary mutation in NRAS.

### AKT

Activation of AKT3 in response to BRAF inhibitor PLX4720 or BRAF siRNA was implicated in the resistance of primary three-dimensional cultures of melanoma cells to BRAFV600E inhibitors [195]. A novel mutation of AKT1 was observed in a resistant tumor [196].

ERK activation, independent of classical RAF/MEK/ERK pathways, was shown to occur in resistant lines with mutant BRAF. PI3K pathway activation could be responsible for ERK activity. Inhibition of either PIK3CA or ERK slowed the growth of these lines selected *in vitro* for resistance to PLX4720 [197]. Targeting ERK activation is a promising strategy to overcome resistance to BRAF and MEK inhibitors. A new ERK inhibitor, SCH772984, is active in nanomolar concentrations *in vitro* and inhibited tumor growth *in vivo*. More importantly, SCH772984 was able to overcome resistance to BRAF or MEK inhibition in cells and tumors that became resistant by acquiring mutations in NRAS or MEK1, respectively. SCH772984 was able to suppress growth of melanoma cells engineered to contain amplified BRAFV600E and even growth of melanoma lines selected for double resistance to BRAF and MEK inhibition [198].

### MAP3K8/COT

In a massive functional kinome screening study, a kinase known as MAP3K8/COT was implicated in both inherent and acquired resistance to BRAFV600E inhibitors. COT is a MAPK independent of RAF and is expressed highly in inherently resistant melanoma or in cells from patients with acquired resistance. Its expression drives resistance to BRAF inhibitors *in vitro* and occurs in relapsing patient tumors [199]. COT-mediated resistance to a RAF inhibitor cannot be overcome by a MEK inhibitor, suggesting that activation of ERK in these tumors occurs outside of the classical RAF-MAPK-ERK pathway. COT might be a direct ERK phosphorylating kinase.

MEK1 mutation C121S was identified in a patient treated with vemurafenib [200]. This mutation was shown to increase kinase activity of MEK1 and confer resistance to both BRAF and MEK inhibition. Two new mutations of MEK, E203K and Q56P, were found in tumors that developed resistance to vemurafenib [187].

RND3

An *in vitro* study with a single BRAF-mutant melanoma line showed that BRAF-inhibitor treatments were associated with reduced expression of RND3, an antagonist of RHOA activation, and elevated RHOA-dependent signaling. Restoration of RND3 expression or RHOA knockdown attenuated the migratory ability of residual cells without affecting overall cell survival [201].

### IRS1

Insulin receptor substrates 1 and 2 (IRS1/2) mediate oncogenic signaling from IGF-1R and are upregulated in melanoma cell lines resistant to BRAF inhibitors or derived from patients that developed resistance to vemurafenib treatment. A new class of drugs that promote phosphorylation and degradation of IRS1/2 were also able to overcome acquired resistance to vemurafenib *in vitro* [202].

### New candidate genes involved in acquired resistance to BRAF inhibition

A CRISPR-Cas9 knock-out screening identified additional candidate genes whose ablation conferred resistance to BRAF inhibitor in melanoma cell line, including neurofibromin 2 (NF2), Cullin 3 E3 ligase (CUL3), and members of the STAGA histone acetyltransferase complex (TADA1 and TADA2B). Previously identified NF1 was a top hit in this screen [203]

### MAPK and PI3K adaptive alterations as predominant resistance patterns

Recently, large-scale studies of the mechanisms of resistance to BRAF inhibitor were performed. In one of them, analysis of 100 biopsies from relapsed melanoma identified MAPK alterations in 70% of patients, and 22% had acquired alterations in PI3K pathway [204], including a novel mutation in the pleckstrin homology domain of AKT1[196]. The heterogeneity within individual melanoma tumors contributed significantly to acquired resistance.

In other studies, mutations in MAPK patwhay were identified in 51% of relapsed tumors from 45 patients treated with BRAF inhibitors. These included mutations in MEK1, MEK2 and in MITF; and in three cases multiple resistance conferring mutations within same tumor were observed [205].

Dual “pre-emptive” inhibition of MAPK and PI3K pathway was suggested as a strategy to induce more durable responses in melanoma. Interestingly, a proteomic study of cellular responses has identified DNA damage response signaling activation in response to dual inhibition of these pathways [206]. Among the most prominent responses were activation-related phosphorylation of ATM, and DNA-dependent protein kinases. Inhibition of these kinases *in vitro* enhanced cell death induced by MAPK and PI3K inhibitors [206].

A trial of a combination of pan-PI3K inhibitor PX-866 and vemurafenib (NCT01616199) is ongoing; preliminary results indicate a significant activity, including in patients previously treated with vemurafenib or trametinib. Sixty-two percent of the patients (8 of 13) who had not previously received treatment with either a BRAF inhibitor or a MEK inhibitor responded to the novel combination.

### Factors that mediate acquired resistance to MEK inhibitors (Table 4)

Similar to the adaptation of BRAF-mutant melanoma cells to BRAF inhibition, resistance to MEK inhibitors could involve multiple pathways. In a study of triple-negative breast cancer treated with a MEK inhibitor, it was found that inhibition of MEK, similar to inhibition of BRAFV600E in melanoma, induces a reprogramming of RTK activation [207]. It is possible that activation of RTK when MEK is acutely inhibited is also involved in responses of melanoma to MEK inhibition, though this has not been shown yet.

### Mutations in MEK1 and MEK2

*De novo* mutations in MEK1 were strongly implicated in resistance to MEK inhibitor selumetinib/AZD6244, both in treated cell lines and in a metastatic tumor from a relapsed patient [208]. Mutations in selected lines resulted in constitutive ERK phosphorylation and higher resistance to MEK inhibitors, but also conferred cross-resistance to mutant BRAF inhibitor PLX4023. Variant MEK1(P124L) was identified in a resistant metastatic focus that emerged in a melanoma patient treated with selumetinib.

### PI3K-AKT pathway

A study *in vitro* has identified basal and treatment-induced activation of the PI3K-AKT pathway as a critical regulator of selumetinib sensitivity in BRAF-mutant cutaneous melanoma cells. Sensitive, but not resistant lines showed upregulation of PTEN expression by selumetinib. Combination of a MEK inhibitor with inhibitors of Akt, mTOR, or IGF1R was able to overcome resistance to MEK inhibitors [209].

### Factors that mediate acquired resistance to BRAF and MEK inhibitors (Table 4)

MEK2 mutation Q60P was identified in a patient that developed resistance to trametinib [210]. Interestingly, the patient's progression tumor also acquired amplification of BRAFV600E. A xenograft tumor derived from a second patient resistant to the combination of BRAF and MEK inhibitors contained identical changes. These cells were resistant to combination treatment with dabrafenib and trametinib, but responded when mTOR inhibitor was added [210]. The mechanisms of resistance to dual inhibition of MAPK pathway by vemurafenib or dabrafenib and trametinib are only beginning to emerge because relatively few patients were treated with this combination in the clinical trials.

Alterations in MAPK patwhay were confirmed in tumors of several other patients treated with dabrafenib and trametinib. Three of these patients also had acquired mutations in MAPK patwhay including a MEK2 mutation [211]. These data suggest addictive dependence of melanoma on MAPK signaling, and a possibility of intervention at the level of inhibition of ERKs.

Inherent resistance, partial and low durability responses, and acquired resistance are major problems in the clinical development of targeted therapies. Recent results of clinical studies combining two targeted therapies have confirmed the long-held belief that combinatorial approaches have significant advantages over monotherapy [26, 166]. The obvious limitation of these studies is that they were, by necessity performed *in vitro*, and their results might not necessary translate into clinical practice. Nevertheless, in a large scale study of 150 small molecule inhibitors in a panel of 28 early passage melanoma lines[39] analysis showed that the most effective drug combination for BRAF-mutant lines was that of vemurafenib, EGFR, and AKT inhibitors, which was cytotoxic even in lines with primary resistance to vemurafenib or in lines selected for resistance to vemurafenib.

### IMMUNOTHERAPY

Malignant melanoma is a tumor extensively investigated as target for immunotherapy for reasons including its high immunogenic potential and the availability of tumor infiltrating lymphocytes (TILs) reactive against melanoma antigens [212]. Earliest approaches had employed active immunization against the constantly growing repertoire of melanoma regression antigens [213], but enthusiasm subsided because of the very modest objective responses to immunization, mostly obtained for low volume disease [214]. The puzzling question of why hasn't successful immunization, generating tumor-cognate lymphocytes, been yielding measurable tumor responses led to the recognition of the inhibitory role of immune check point modulators expressed on melanoma cells or on activated lymphocytes [215, 216]. Understanding the role of costimulation opened a new era in the clinic and an intense search for additional targets for immunotherapies with improved efficacy to toxicity ratio.

Interleukin-2 was the earliest immunotherapy for metastatic melanoma, approved by the FDA in 1998, followed by interferon-α2b as an adjuvant therapy and ipilimumab (anti-CTLA-4 antibody) as therapy for advanced disease, approved by the U.S. Food and Drug Administration (FDA) for melanoma in March 2011. While the response rates to IL-2 and ipilimumab given as a single agent are in the range of 10% to 20% [217, 218], when induced, responses to IL-2 and ipilimumab are typically long lasting and sometimes complete. The reasons for lack of response to IL-2 are starting to emerge: a recent study found that the T cell subset most induced by IL-2 in melanoma patients is regulatory T cells (Treg) expressing positive for CD4, CD25, Foxp3 and the inducible T cell costimulator (ICOS). The high levels of these ICOS expressing peripheral Tregs was a strong predictor of the lack of response to IL-2 [219].

In recent years, investigational agents have included adoptive T cell transfer, blocking antibodies against inhibitory immune molecules, stimulatory antibodies for immune cells, and immunization with distinct cancer cell antigens. Recently, interest in the role of mutated antigens being more immunogenic and therefore triggering a more robust immune response, has risen [220]. Identification and validation of biomarkers predictive of responses to immunotherapy is a rapidly developing field (reviewed by Ascierto et al. [221]). These might include a variety of tumor and tumor microenvironment–specific alterations, the immune parameters of the individual patient. The mutational load of a tumor could be also a predictive factor, as it is plausible that highly mutated tumors are more immunogenic, therefore triggering a more robust immune response.

In light of the complexity of the immune response, it is very likely that combining immunotherapies will be necessary to induce better clinical responses, but even at this point it is clear that there is a life-prolonging effect of immunotherapy in many patients and a potential for cure.

### Immunomodulatory antibodies

The use of immune checkpoint inhibitors (antibodies that block proteins inhibiting immune response) has gained particular interest after approval of ipilimumab/anti-CTLA4 antibody for treatment of melanoma in 2011, and more so after the recent reports on the clinical efficacy of antibodies blocking PD-1. Table 5 shows some of the clinical trials with immune-system-targeting antibodies, which are described in more detail below.

**Table 5:** Antibody-based immunotherapy of melanoma

Target	Desired effects	Antibodies	Trials
CTLA-4	Relieve the immune checkpoint	Ipilimumab/Yervoy	Approved
PD-1	Relieve the immune checkpoint	MDX-1106, CT-011, MK-3475	NCT01024231, NCT01176461, NCT01721746, NCT01621490, NCT01721772, NCT01783938, NCT01714739, NCT01435369, NCT01295827, NCT01704287
PD-L1/B7-H1	Relieve the immune checkpoint	MDX-1105-01, MPDL3280A, MEDI4736	NCT00729664, NCT01633970, NCT01375842 NCT01693562
4-1BB/CD137	Stimulate T cells	BMS-663513	NCT01471210
CD40	Stimulate T cells	CP870,893	NCT01103635
GITR	Inhibit T regs	TRX518	NCT01239134
OX40/CD134	Stimulate T cells	Anti-OX40	NCT01689870

CTLA4 is an inhibitory molecule expressed on T cells that is involved in the negative regulation of the T cell interaction with antigen-presenting dendritic cells (APCs). CTLA4 inhibits binding of CD28 on T cells to B7 proteins on APCs, thus weakening the costimulation of effector T cells. In addition, CTLA4 is expressed on regulatory T-cells (Treg), and the immune effects of anti-CTLA4 may be a combination of enhancing effector T-cell function while blocking Treg [222]. CTLA4 is also expressed on tumor cell lines [223] and in human melanoma [224]; blockade of CTLA4 *in vitro* induces apoptosis of melanoma cells, indicating that CTLA4 might have non-immune functions. Available results from clinical trials indicate that the response rates to CTLA4 blockade with human monoclonal antibodies ipilimumab and tremelimumab are at most 18%, but overall survival is superior to what is seen with cytotoxic therapies. Significant immune toxicities were reported in a number of trials, and, interestingly, strong association of immune-related toxicities and responses were observed (reviewed by Flaherty et al. and Sapoznik et al. [8, 225]).

Potential markers that could predict response to ipilimumab are of obvious importance. Clinical trial NCT00261365 incorporated investigation of a number of parameters in tumor biopsies pre- and post-treatment with ipilimumab. Significant associations were detected between clinical activity and high-baseline expression of FoxP3 and indoleamine 2,3-dioxygenase (IDO), and an increase in tumor-infiltrating lymphocytes (TILs) [226]. The immunosuppressive role of IDO in the context of immunotherapies was later shown in the B16 murine melanoma model [227]. IDO catalyzes tryptophan degradation and inhibits T cell function. Inhibitors of IDO INCB024360 and 1-methyl-D-tryptophan are in several clinical trials for different malignancies, including a randomized phase I-II trial of combination of ipilimumab with INCB024360 (NCT01604889).

Trials of ipilimumab and gp100 peptide vaccine—each as a single agent or in combination—showed limited responses, but the responses were long lasting. Early-phase combination trials with ipilimumab include the following second agents: bevacizumab, high dose interferon α-2b, IL-2, GM-CSF, anti-PD-1 antibody (see below), antibody to NK receptor KIR BMS-986015 (in patients with melanoma or other tumors), immunostimulatory cytokine IL-21, and even an oncolytic herpes simplex 1 virus (talimogene laherparepvec/T-VEC) —designed to replicate selectively in tumor cells and to express GM-CSF (the virus preparation is injectable directly into tumors) —as well as a variety of chemotherapeutic agents and surgical/radiotherapy interventions (Supplementary Table 2). Reported results from trial NCT01134614 combining ipilimumab with GM-CSF showed an increased OS in the combination arm (ASCO abstract #CRA9007, 2013).

PD-1, like CTLA-4, is an inhibitory receptor; however, its expression is not limited to T cells and is found in B cells and some myeloid cells. The PD-1 ligands PD-L1 and PD-L2 have different expression patterns, with PD-L1 found on multiple normal and cancerous cells including melanoma tumors, where it provides, once bound by PD-1, peripheral tolerance to “self” antigens [228]. PD-L2 is expressed on APC cells, providing tolerance to orally administered antigens. Interactions between PD-1 and its ligands attenuate immune responses [229], and, in the context of cancer, serve to protect tumor cells from cytotoxic T cells. At the same time, PD-1 is expressed on CD8+ T cells in patients with metastatic melanoma—particularly within the tumor microenvironment where they encounter chronic antigen exposure [230], suggesting that the immune response to melanoma is inhibited under these circumstances.

Humanized antibodies to PD-1 and PD-L1 have been developed and tested in clinical trials for several cancers including melanoma. Anti-PD1 antibody nivolumab was well tolerated, and the maximum tolerated dose has not been reached [231]. This study also found a strong correlation between pretreatment tumor expression of PD-L1 and responses, with a 36% response rate in patients whose tumors expressed PD-L1, and 0% in the PD-L1-negative group. However, a larger study of PD-L1 expression as a biomarker of response in solid tumors including melanoma reported that the correlation is not as solid as reported, because some patients with PD-L1 negative tumors had clinical responses to nivolumab (http://meetinglibrary.asco.org/content/113904-132). This may be because PD-L1 expression in the tumor is dynamic and is upregulated by factors such as the production of gamma interferon by infiltrating T cells. The better safety profile of PD-1 antibody versus CTLA-4 antibody is most likely due to the fact that the latter targets a peripheral interaction between T cells and tumor or APCs. Inhibition of PD-1 probably inhibits peripheral interactions as well, through PD-L2 on APCs, but it could be expected to act more locally at tumor sites by preventing inhibition of tumor-infiltrating PD-1-expressing T cells through interaction with PD-L1 on tumor cells.

### Clinical trials targeting PD-1/ PD-L1 interaction

Preliminary results from an early-phase clinical trial with anti-PD1 antibody BMS-936558 (MDX-1106, ONO-4538, nivolumab) showed durable responses in 28% of patients with melanoma [232]. Results a from completed trial reported that 1- and 2-year survival rates were 62% and 43%, respectively [233]. Currently there are at least eight trials ongoing with nivolumab for melanoma.

Two other anti-PD-1 antibodies (CT-011: CureTech Ltd. and MK-3475/lambrolizumab: Merck) are also in clinical trials. Results of a clinical trial of lambrolizumab in advanced melanoma produced very promising results. Of 135 patients treated, 38% had durable responses as evaluated by RECIST. Remarkably, patients that have received ipilimumab prior to enrolling in this trial had a similar response rate. Importantly, lambrolizumab showed a favorable safety profile [234].

An anti-PD-L1 antibody (MPDL3280A/BMS-936559/MDX-1105 is being tested as well. Preliminary results with anti-PD-L1 antibody showed significant tumor shrinkage in 21% of 140 patients who had a variety of cancers; 9 out of 52 melanoma patients had objective and durable [235]. Recent preliminary results of MDPL3280A trial showed objective responses in 28% of patients with melanomas of different origins (45 patients total), but none in 4 patients with uveal melanoma. Trial NCT01656642 examines combination of MDPL3280A with vemurafenib.

An important study has shown that combining blockade of both immune checkpoints (PD-1 and CTLA4) is highly synergistic in rejection of melanoma tumors in an animal model through strong stimulation of effector T cells and IFN-g production [236]. Anti-PD-1/nivolumab/MK-3475 and anti-CTLA-4/Yervoy antibodies are being combined in clinical trials with the hope of synergistic effects (NCT01024231). This combination treatment, when administered concurrently to 52 patients at a variety of dose levels, produced an objective response rate of 40% with 5 complete responses. The degree of observed tumor shrinkage was more than 80% for most of responding patients. In several patients in the study, tumors disappeared completely, as could be determined by imaging. Grade 3 or 4 adverse events related to therapy occurred in 53% of patients, but were generally reversible [237]. A randomized phase III trial NCT01844505 for ipilimumab + nivolumab with the endpoint of OS is recruiting melanoma patients. Phase II trial NCT01783938 will explore sequential administration of nivolumab and ipilimumab. Recent results showing responses to nivolumab in patients who progressed on ipilimumab treatment support the schedule of sequential administration of these antibodies [238]. Ipilimumab-refractory and naïve patients had a similar response rate of 25% in this trial.

In general, a concept is emerging that different immune checkpoints might have non-overlapping functions in immune escape; therefore, targeting more than one inhibitory molecule might be a general strategy for future approaches to immunotherapies. In addition to CTLA and PD-1/PD-L1, other proteins (LAG-3 and TIM-3) act as checkpoints. In addition, several proteins listed below are known to positively modulate T cells function. There are dozens of molecules now known to upregulate or downregulate immune responses and future trials evaluating agonists and antagonists of these molecules in combination may be promising.

4-1BB. Also known as CD137, 4-1BB is a member of the TNF receptor superfamily, and is an activation-induced costimulatory molecule. Binding of 4-1BB by its ligand or antibody induces powerful CD8+ T-cell activation, IFN-γ production, and cytolytic activity. An agonistic antibody, BMS-663513 (Bristol-Myers Squibb) was in clinical trials for melanoma and other tumors. Safety concerns caused suspension of the trial after several patients developed liver problems, including high-grade hepatitis. However, the antibody has promise in expanding the repertoire of functional CD8+ effector cells during T-cell expansion for autologous cell transfer (ACT). A recent study showed that addition of BMS-663513 to the expansion cultures of T cells isolated from metastases strongly increased the frequency and yield of CD8+ cells as well as their cytotoxic activity *in vitro* [239].

GITR is a co-stimulatory receptor expressed after T-cell activation that enhances T-cell function and survival. Importantly, GITR also negatively affects regulatory T cells (Tregs), and treatment with GITR agonistic antibody destabilizes intra-tumor Tregs allowing for more efficient cytolysis by CD8+ T cells [240]. A trial with anti-GITR antibody TRX-518 is ongoing (NCT01239134).

OX40 is not involved in effector T-cell activation, but rather, promotes T-cell survival and expansion. In a clinical study, based at the Portland Providence Medical Center in Oregon, patients received three infusions of the agonistic mouse anti-OX40 antibody within a week. The xenogeneic nature of the antibody precluded further treatments. Nine of 27 patients experienced minor tumor shrinkage, although none met RECIST (response evaluation criteria in solid tumors) criteria for objective responses.

CD40. Unlike the co-stimulatory targets above, CD40 is expressed on APCs, while its ligand is expressed on T cells. Binding of the two acts as a powerful enhancer of APCs' ability to present antigens and activate T cells against foreign targets [241]. A number of cancer patients with diverse solid tumors have received infusions of agonistic antibody CP870,893, often in combination with conventional chemotherapy, making it difficult to evaluate the antibody contribution. In those given antibody alone some objective responses were observed, particularly in a few patients with melanoma. A surprising finding was that treatments did not increase numbers of TILs in the tumors. In a mouse model, antibody treatments induced an influx of macrophages into tumors, presumably with enhanced cytotoxic activities. A phase I trial NCT01103635 combining CP870,893 with tremelimumab in melanoma and other tumors is ongoing. A dose escalation trial of CP870,893 is ongoing where antibody is combined with Oncovir poly IC:LC and NY-ESO-1/gp100 NCT01008527.

CEACAM1 is a potential new target for the development of targeting antibodies. CEACAM1 is a carcinoembryonic, antigen-related adhesion molecule 1 from the IG superfamily whose expression is absent from normal melanocytes, but often found in melanomas, particularly those that are metastatic. CEACAM1 was shown to protect melanoma cells from T cells *in vitro*, and, moreover, its expression was found on T cells and NK cells from melanoma patients, presumably enabling a homotypic inhibitory interaction. A mouse antibody to CEACAM has no apparent effect on CEACAM1-expressing melanoma cells *in vitro*, but renders them susceptible to elimination by T cells *in vitro* and in an *in vivo* xenograft model. These findings provide a strong rationale for developing CEACAM1-based therapeutics for the treatment of metastatic melanoma [225].

### Cytokines

Interleukin-2 (IL-2), a protein produced primarily by CD4+ T-cells that activates and induces proliferation of natural killer (NK) cells, CD8+, and CD4+ T cells, is FDA-approved for the treatment of metastatic melanoma. This is due to the long, durable responses lasting over 10 years in a subset of patients. However, objective clinical responses to IL-2 alone are under 15% in most series, and the treatment is related to significant adverse reactions including pulmonary edema, hypotension, fever, and chills. In a randomized clinical trial for metastatic melanoma patients, IL-2 combined with a gp100 peptide vaccine doubled the clinical responses compared to those patients receiving IL-2 alone. The vaccine + IL-2 group also had increased progression-free survival (PFS), but only a trend towards improved overall survival [242]. Currently, a trial with IL-2 in combination with a MAGE vaccine containing an improved adjuvant is ongoing. One interesting small trial combined high-dose IL-2 with ipilimumab in 36 patients with metastatic melanoma [243]. The overall response rate of 25% was most notable because 6 of the 9 responders achieved CRs (CR rate 17%) and all were sustained beyond 6 years. This suggests that combining stimulatory cytokines with checkpoint blockade is a strategy worth further investigation.

Interferon-alpha is FDA-approved for the treatment of stage III melanoma in the adjuvant setting. Interferon has been shown to increase relapse-free survival (RFS)—but in most studies, not overall survival. Recently a weekly pegylated formulation was also FDA-approved for the same indication. Data suggests that patients with ulcerated primaries and microscopic compared to palpable nodal disease are subsets that may benefit most from interferon. Toxicities of interferon include immune hepatitis, fever, chills, and fatigue, and the recommended regimen includes one month of high-dose intravenous therapy followed by 11 months of subcutaneous injections.

Cytokines under investigation for the treatment of metastatic melanoma include IL-21, which produced an ORR of 22.5% [244], and is tested both alone and in phase I trials in combination with anti-CTLA4 (NCT01489059) and anti-PD1 antibody (NCT01629758). Trials of IL-12 in melanoma, NCT01397708 and NCT01502293, that deliver various formulations of IL-12 systemically or intratumorally, have shown some positive responses.

### Adoptive cell transfer

Adoptive cell transfer (ACT) involves the selection of autologous lymphocytes with antitumor activity, their expansion/activation ex vivo, and their reinfusion into the patient, often in the context of lymphodepleting regimens to minimize endogenous immunosuppression (reviewed by Galluzzi et al. and Itzhaki et al.[245, 246]). T-cell-adoptive therapy for metastatic melanoma has been quite successful in achieving objective regressions in about 50% of patients [247] and produced durable complete regressions. Despite the clear therapeutic efficacy in some melanoma patients with ACT, there are a number of challenges for this approach to become widely accepted and FDA approved, including the complex methodology required to manufacture large numbers of T cells, which has restricted this therapy to a limited number of patients in a handful of centers. Increased utilization of this approach will require a simplification and improvement of the methods for T-cell expansion and a modification of the treatment regimen associated with decreased toxicity such as the use of lower doses of IL-2. Importantly, while it is clear that patients failing anti-CTLA4 can respond to ACT, future studies will determine whether the same is true for patients who fail anti-PD1 therapy.

Different aspects of ACT are being examined, among them the role of cytokines such as IL-15 and T-cell-produced IL-9 in T-cell-based therapy [248]; concomitant low-dose IL-2 in patients treated with ACT [249]; facilitation of the long-term engraftment of T cells by using the memory-T-cell population [250, 251]; the basis for the relapses frequently observed after ACT immunotherapy, such as inflammation-induced, reversible loss of melanocytic antigens mediated by TNF-a [252]; the immunosuppressive role of Tregs and myeloid-derived suppressor cells (MDSC) [253]; and the nature of the relevant tumor-rejection antigens being targeted. The potential of using the presence of the immunostimulatory marker CD137 on CTL for selection of tumor-reactive T cells was successfully explored recently [254].

Analysis of the repertoire of T cells in the successful ACT treatments of three melanoma patients through exomic sequencing of tumors established a correlation between the ability of infused T cells to recognize specific mutated proteins present in tumors. Candidate mutated epitopes were identified using major histocompatibility complex–binding algorithm for recognition by TILs [220]. In one patient successfully treated by ACT in a previous trial, the specific T cells recognizing a mutated epitope persisted in peripheral blood for over 5 years [255].

A recent phase II clinical trial reported objective clinical responses in almost half of the 31 patients enrolled, with two complete remissions and a significant increase in PFS [256]. The study also analyzed characteristics of infused T cells associated with significant responses, and found significant correlations with a higher number of TIL infused: a higher proportion of CD8+ T cells in the infusion product; a more differentiated effector phenotype of the CD8+ population; and, unexpectedly, a higher frequency of CD8+ T cells co-expressing the negative co-stimulation molecule “B- and T-lymphocyte attenuator” (BTLA). A phase I/II trial is investigating the use of autologous TILs enriched for CD8 and engineered to secrete IL-12, which will be infused after patients undergo a myeloablative regimen of chemotherapy (NCT 01236573). On the other hand, a randomized trial suggested that use of unselected young autologous T cells is preferable to enrichment for CD8+ cells because the latter are more laborious to prepare and did not offer a therapeutic advantage [257].

Failure of the infused CD8 cells to persist in the patients could be a major cause for the limited efficacy of TIL approaches to treatment. In a number of trials this was addressed by administration of IL-2 after infusion, or lymphodepletion prior to infusion. To increase the persistence of infused T cells in patients, an approach was tested to stimulate CD8+ cells *in vitro* with MART-1-presenting APCs in the presence of IL-2 and IL-15. These CTLs displayed a memory phenotype, both central and effector, trafficked successfully to the tumor sites, and produced a complete response in one of nine patients, as well as several partial responses and disease stabilization [258].

Another approach to ACT therapy is to create tumor-reactive T-cell populations from PBL by retrovirally transducing them with chimeric antigen receptors (CAR) to tumor-associated antigens or natural T-cell receptors against antigens presented in the context of MHC. CAR-modified T cells do not depend on MHC-mediated antigen presentation, which is frequently dysfunctional in tumors. The steps and considerations in producing T cells with CARs and their advantages as well as pitfalls were recently reviewed [259]. In melanoma, T cells redirected to recognize MART-1 have produced significant clinical responses (reviewed by Strauss [260]). A tumor-testis antigen on melanoma and other tumors, NY-ESO-1, has been effectively targeted with an MHC-restricted T-cell receptor in another trial[261].

Some remarkable responses were observed in individual patients treated with CD4+ T cells. For example, autologous transfer of expanded CD4+ T cells recognizing melanoma antigen NY-ESO-1 produced a complete and durable response in one patient trial [262]. Recent findings indicate that CD4+ T cells could also contribute to the success of ACT by inducing tumor senescence through production of high levels of IFN-γ and TNF [263].

### Dendritic cell–based immunotherapy

Dendritic cells (DCs) are the most potent-presenting cells in the immune system; clinical efforts to use DC to induce potent anti-melanoma immune responses have been ongoing for at least a decade [264]. However, as with most immunotherapies, the responses to DC-mediated vaccination have been unpredictable and variable.

Stimulation of anti-melanoma responses by DCs involves a number of steps that need better scientific understanding and clinical exploration. To arm DCs with the antigens needed to be presented to immune effector cells, DC are usually “loaded” with antigens expressed in tumors by incubation with the tumor antigens and an adjuvant ex vivo. This process induces maturation of DCs, which involves processing the antigens via proteasomal degradation and presenting them to T cells on a variety of MHC-family molecules. As so-called “professional antigen presenting cells”, DCs can present antigen to and efficiently co-stimulate both CD4+ cells and CD8+ cells. The T-helper cells could stimulate B-cell-based humoral responses, and killer T cells could have direct cytotoxic activity against antigen-presenting tumor cells. DCs can also react to as well as stimulate the components of the innate immune system such as NK cells and phagocytes. Yet other DC interactions can also induce regulatory T cells that dampen immune responses. Finally, DCs are also influenced by a large variety of soluble factors, many of which are abundant in the tumor microenvironment.

A number of variables remain to be fully explored in DC vaccination: the source of DCs, the optimal methods for maturation ex vivo, the route of introduction into patients, the ways to overcome the tumor-generated immunosuppressory activities, and more.

Clinical trials have explored DCs generated by different methods and injected by different routes in patients with metastatic melanoma (only a few are mentioned here). DCs can be isolated from peripheral blood and from Langerhans cells—a subset of DCs present in skin—or other sources (see below). For example, an early clinical trial that concluded in 1998 produced promising results. Patients were vaccinated with monocyte-derived DCs pulsed with tumor lysate or a mix of tumor-related peptides, and stimulated with GM-CSF and IL-4. Five of 16 patients had objective responses [265]. Monocyte-derived DCs were pulsed with a single tumor peptide from Mage-3A1 and a recall antigen in another study. Evaluation of immune responses showed that 8 of 11 patients had an expansion of Mage-3A1-recognizing CD8+ T cells, and 6 patients showed regression of some metastases, but no formal PRs or CRs were achieved [266]. It was noted that some nonregressing tumors lacked Mage-3A1 expression and specific T cells.

In a different approach, immature DCs were generated from CD34+ hematopoietic progenitor cells through stimulation with a predefined cytokine cocktail, and matured *in vitro* by pulsing with a pool of peptides derived from known melanoma antigens [267]. This study reported durable immune responses and perhaps one objective clinical response.

Langerhans cells were directly compared to monocyte-derived DCs in a clinical trial, and were found to be similar in terms of eliciting immune responses, but different in terms of the need for cytokine stimulation [268]. The role of the route of injection was explored in a study that injected monocyte-derived, stimulated DCs either intranodally or intradermally. The latter produced very low percentages of cells surviving and migrating to lymph nodes [269], but the induced immune responses (stimulation of tumor-specific T cells) were superior in the patients receiving intradermal inoculation [270].

Stimulation of DCs could be performed not only by exposing them to tumor antigens, but also by transfection of mRNA coding for the antigens [271]. The latter might have an advantage of longer exposure and correct processing of the antigens. Indeed, it was shown to produce strong immune responses after intranodal injection, inducing a broad repertoire of IFN-γ producing TAA-specific CD8+ and CD4+ T cells [272]. Stage III melanoma patients in this study also had significant immune responses.

A recent study reported a further significant improvement on DC maturation ex vivo. Mature DCs (used for vaccination) express a specific type of proteasomes, the immunoproteasomes (iPs) that are responsible for antigen processing in immune cells. However, tumor cells express constitutive proteasomes (cPs) that contain three different subunits. It was hypothesized that generating T cells against epitopes from one type of proteasome (iPs) might not result in recognition of epitopes generated by target cell proteasomes (cPs), preventing optimal recognition and immune attack of tumor cells. Therefore, the patient-derived DCs were transfected not only with the RNA for tumor antigens, but also with shRNA, which enforced the default use of only cPs in the DCs. Patients vaccinated with cPs-expressing DCs had lower levels of circulating melanoma cells and enhanced melanoma-directed T-cell-lytic activity, which was of a longer duration compared to patients in control arms of the study. Of the five patients vaccinated with cPs-expressing DCs, one had a complete response, and one had a partial response [273].

Another important component of successful vaccination with DCs is their ability to stimulate CD8+ T cells, which requires production of IL-12-p70 by DCs, which, in turn, depends on stimulation of DCs by two signals: CD40 or TLR and INF-γ A recent small clinical trial used DCs from peripheral blood mononuclear cells (PBMCs) of melanoma patients pulsed with gp100 peptides. In addition, maturation *in vitro* included stimulation of DCs from PBMCs with both CD40L and INF-γ. The study of seven patients showed a straightforward correlation between the clinical responses and levels of IL-12 produced by their DCs. The one patient with complete remission had the highest levels of IL-12. Moreover, the study has identified the deficiency in nonresponding patients as the inability to produce IL12 without additional stimulation with TLR agonist. Addition of poly I:C to the maturation protocol has rescued production of IL-12 by these DCs *in vitro* and improved the clinical outcomes [274].

Although combination immunotherapies involving DCs and immunomodulatory antibodies may hold promise [275], overall clinical results with DC vaccines remain sporadic, anecdotal, and inconsistent. Clinical investigators continue to struggle with an overwhelming number of variables, reflecting the complicated biology and role of this immune cell.

### Adoptive transfer of natural killer cells

NK cells are an essential part of the innate immune system and have been considered in immunotherapies of melanoma. NK cells in principle are able to recognize and destroy virally infected or malignant cells without a need for antecedent antigenic stimulation. However, their activity, as with other immune effector cells, also depends on balance of stimulatory or inhibitory factors. NK cells themselves are regulated by inhibitory receptors, one of which, KIR (killer-cell immunoglobulin-like receptors), is being targeted in a clinical trial with the antagonistic antibody BMS-986015 that is designed to block the inhibition by KIR of tumor-cell cytolysis.

NK cells can be obtained from PBMC where they comprise 5% to 15 % of the lymphocyte population, expanded and activated *in vitro* and adoptively transferred. In fact lymphokine activated killer cells (LAK cells) given in large numbers 20 years ago were largely IL-2 activated NK cells [276]. These prior trials, as well as a more recent trial combining autologous NK transfer with preparative lymphodepletion, have not demonstrated benefit attributable to the NK cells [277]. New strategies might include combination of NK transfer with immunomodulatory antibodies or even transfer of activated allogeneic NK cells [278]. The cytokine-induced killer cells (CIKs), which can also be isolated from PBMCs, were recently characterized as a variant NK subtype that have a cytotoxic activity against autologous melanoma cells [279].

### Influence of targeted therapies on the responses to immune therapy

### BRAF/MAPK signaling and TIL

Activated BRAF/MAPK signaling was shown to be essential in the development of immune evasion in melanoma [280]. Prior to clinical development of BRAF and MEK inhibitors, it was shown that inhibition of the MAPK pathway leads to increased expression of melanocytic antigen expression, including MART-1, gp100, and tyrosinase; whereas enforced expression of mutant RAF downregulated expression of these antigens [281]. Mutant BRAF induces expression of IL-1α in stroma, also leading to immune suppression [282]. In a mouse model, BRAFV600E induced expression of CCL2, an immunosuppressive chemokine, while PLX4720 treatment downregulated tumor CCL2 gene expression and increased numbers of CD8 TILs [283].

There were concerns that treatment with BRAF/MEK inhibitors might have a direct inhibitory effect on T-cell function and it was indeed shown later that treatment with MEK inhibitors, but not BRAFV600E inhibitors, impairs T-lymphocyte function [284].

A number of subsequent reports have confirmed that inhibition of mutant BRAF could potentiate immune responses in melanoma, suggesting that blockade of immune checkpoints in combination with BRAF inhibitors could have clinical value. Treatment with BRAF inhibitors vemurafenib and dabrafenib resulted in markedly increased numbers of TILs in tumor biopsies obtained pre- and post-treatment in 15 patients [285]. Another study found that dabrafenib has no detectable negative impact on existing systemic immunity or the *de novo* generation of tumor-specific T cells in patients [286].

A single report claimed that selective BRAF inhibition decreases tumor-resident lymphocyte frequencies in a genetically engineered mouse (GEM) melanoma model; moreover, treatment with ipilimumab did not restore the numbers of TIL [287]. Although GEM models are clearly helpful in studies of targeted therapies, their utility in the evaluation of immunotherapies is not clear. It is likely that melanoma's immunogenicity depends on the high number of missense mutations inducing antigens that are recognized as “foreign” by the immune system, but this feature is not reproduced in most of the GEM models.

The reported analysis of biopsies taken before and after treatment with BRAF or BRAF+MEK inhibitors showed the following: enhancement of melanoma antigen expression, increase in CD8+ TILs, and decrease in immunosuppressive cytokines IL-6 and IL-8. Interestingly, inhibitory PD-1 was increased on T cells and its immunosuppressive ligand PDL1 was also increased with BRAF inhibition. This supports the hypothesis that targeting inhibitory immune interactions may be critical in augmenting responses to BRAF-targeted therapy in patients with melanoma [288].

### Adoptive cell transfer therapy and targeted therapies

Adoptive cell transfer therapy could also be of more benefit when combined with vemurafenib, as a mouse model suggests [289]. In a recent study, vemurafenib increased MAPK signaling, *in vivo* cytotoxic activity, and intratumoral cytokine secretion by adoptively transferred cells (T cells genetically engineered to recognize tumor-expressing antigens). Another study reported that administration of PLX4720 significantly increased tumor infiltration of adoptively transferred T cells *in vivo* and enhanced their antitumor activity in a mouse model. Apparently, PLX4720 negatively affected expression of VEGF by tumor cells. Importantly, analysis of human melanoma biopsies before and during BRAF inhibitor treatment also showed downregulation of VEGF consistent with the preclinical murine model [290].

An *in vitro* study analyzed effects of BRAF and MEK inhibition on function of dendritic cells (DC) *in vitro* and found that BRAF-mutant melanomas suppress immune function of dendritic cells. Inhibition of BRAF, but not MEK could reverse suppression of DC function. As has been reported for the effects of MEK inhibition on T cells [284], inhibition of MEK did negatively affect DC function and viability. Vemurafenib, but not MEK inhibitors, was therefore suggested as a preferable candidate for combination immunotherapy approaches in BRAF-mutant melanoma. [291].

Another *in vitro* study examined how vemurafenib affects the ability of the TILs to recognize autologous BRAF(V600) mutant melanoma cell lines *in vitro* [292]. The results showed a significant increase in recognition of the inhibitor-treated melanoma cells, attributed to increased expression of MHC class I-associated proteins and heat-shock proteins.

These studies in general provide a strong rationale for combination therapies involving BRAF inhibition with immunotherapies involving either antibodies to immunosuppressive molecules or adoptive cell transfer. Several trials are ongoing, but one has been closed for toxicity. The phase I trial of concurrent vemurafenib and ipilimumab (NCT01400451) was closed in March 2013, due to dose-limiting liver toxicities [293]. This highlights the need for careful evaluation of combination strategies, in particular because the drugs in this trial were approved, and had little overlap in terms of side effects. Other trials will evaluate combinations of vemurafenib and anti-PD-L1 antibody MPDL3280A (NCT01656642), vemurafenib and HD-IL2 (NCT01683188), vemurafenib and adoptive cell transfer (NCT01585415), HD-IL2 + adoptive cell transfer + vemurafenib (NCT01659151), and more. Whether to give targeted therapy or immunotherapy first to patients with BRAF mutant melanoma remains an important clinical question. Patients with rapidly progressing disease may need to start with BRAF inhibitors since this treatment works quickly. On the other hand, if the disease is not rapidly progressing, beginning with immunotherapy makes sense due to the durability of response in some patients.

Mutant BRAF and/or its inhibition are not expected to influence outcomes of all immune therapies. For example, a recent trial showed that treatment with IL-21 produced an overall response rate of 22.5% in melanoma and responses were not related to the status of BRAF [244]. Treatment with high-dose IL-2 was more effective in patients with NRAS mutations versus BRAF mutations or WT/WT tumors [294].

Other melanoma-related pathways might also play a role in responses to immune therapy. Wnt/β-catenin signaling might be involved in immunosuppression in melanoma, as high levels of nuclear β-catenin in melanoma cells impaired maturation of DCs at least in part through increased production of IL-10 and inhibited IFN-α production by melanoma-specific CTLs [295]. It is entirely possible and even likely that other oncogenic pathways in melanoma also mediate immune suppression in melanoma.

### Immune environment in melanoma

The role of B cells in immune responses in melanoma.An extensive study measured humoral B-cell responses in melanoma patients, and concluded that on average melanoma patients have highly increased antibody responses against melanoma cells compared to healthy volunteers. Interestingly, the B-cell-mediated immune responses were significantly diminished in patients with metastatic melanoma versus primary disease [296]. The impaired B-cell functionality with loss of CD27+ (memory) cells was previously reported in metastatic melanoma [297].

Intratumoral IgG-producing B cells have been reported in melanoma, but their functional significance was largely unknown. A study published in March 2013 found a skewing of B cell repertoire in melanoma tumors, with highly increased numbers of IgG4-producing B cells relative to normal ratios. High IgG4/IgG total ratios are considered a limiting response by the immune system to contain immune activation. In melanoma, production of B cells secreting inhibitory IgG4 is stimulated by the tumor secreted IL-10 and IL-4. The tumor-specific IgG4 even inhibited IgG1-dependent tumor-cell killing through engagement of the FcγRI effector mechanisms. Moreover, high circulating levels of IgG4 in serum of patients were associated with poor prognosis [298].

Myeloid-derived suppressor cells have been implicated as an important component in suppression of immune responses in melanoma. Higher frequency of these cells in advanced melanoma patients is associated with worse prognosis [299, 300]. Strategies targeting these cells are being developed, including inhibition of CSF-1 receptor [301] to improve the efficiency of ACT in melanoma.

### Immune cell infiltrates in melanoma

The presence of lymph node–like structures in solid tumors has been recognized relatively recently, and is considered to have a prognostic value, in particular as related to immune therapy [302]. It was suggested that the presence of these lymphoid structures could be due to locally tumor-produced chemokines, and could be useful in selection of patients for whom immunotherapies might work best. A large study of the TIL grades in primary melanoma concluded that the high levels of TIL constitute an independent good prognostic marker of survival not related to other clinicopatholigic features [303]. Moreover, a particular 12-chemokine signature produced by tumors was identified by expression profiling, and was shown to be predictive of the formation of these lymph node–like structures. The hope is that this expression signature might be used for selection of patients for whom long-lasting responses to immunotherapy are possible [304]. Recently, the subset of melanoma tumors characterized by the presence of T-cell infiltrates was found to demonstrate activation of three immunosuppressive markers: indoleamine-2,3-dioxygenase (IDO), PD-L1, and FoxP3+ regulatory T cells (Tregs). Moreover, expression of these actually appears to be secondary to the infiltration of CD8+ T cells, implying that the immune system rather than the tumor initiates the creation of immunosuppressive environment [215]. Tregs subset involved in inhibition of cytotoxic T cells is apparently characterized by expression of the chemokine receptor CCR4, and CCR4-based depletion of these Tregs was successfully used to stimulate specific anti-tumors immune response [305]. Nevertheless, these findings still support the notion that tumors with CD8+ T-cell infiltrates could benefit from treatments with immune-modulatory antibodies and likely other immunotherapeutic approaches.

The question then arises about the subset of melanoma patients whose tumors do not contain T cell infiltrates and are thus not likely to respond to immune interventions. It is reasonable to suggest that the microenvironment in these tumors is prohibitive for infiltration of T cells, and seek ways to overcome these tumor properties. A recent intriguing study showed that neoadjuvant local irradiation of tumors promotes recruitment of infused tumor specific CTLs in a pancreatic transgenic model and xenotransplant model of human melanoma. In both models, this led to profound inhibition of tumor progression. The mechanism, investigated in the pancreatic cancer model only, involved stabilization of microvasculature and tumor-infiltrating macrophages and most likely was associated with inflammatory processes triggered by irradiation. These findings raise a possibility of using local irradiation (possible only for macroscopic tumors) or infusion of activated macrophages as means of promoting CTL recruitment into tumors [306].

The oncolytic Newcastle disease virus (NDV), injected intra-tumorally, was shown to induce inflammatory immune infiltrates in not only in injected tumor, but also in distant tumors in a murine B16 melanoma model. Importantly, NDV showed a strong potentiating effect on the efficacy of the systemic CTLA4 blockade, resulting in rejection of pre-established distant tumors. Increase in TILs in the single injected as well in distant tumors was shown to make them susceptible to systemic therapy with immune checkpoint modulatory antibodies. These observations, though limited to a single model, provide a rationale for exploring the potential of oncolytic viruses such as NDV in combination treatments with immune checkpoint antibodies [307].

### METABOLISM AND AUTOPHAGY AS TARGETS IN MELANOMA THERAPY

There have been few attempts to target melanoma metabolism with the exception of several known metabolic preferences listed below. However, recent discoveries, such as high levels of OXPHOS (oxidative phosphorylation) in highly aggressive melanoma mediated by elevated expression of PGC-1a [60], might prompt more interest in targeting melanoma metabolism. For many years, serum lactate dehydrogenase (LDH) levels were used as a predictive factor to identify melanoma patients with worsened prognosis, and they presumably are indicative of the high glycolytic character of tumors, though the energy requirements of melanoma might be fulfilled through a more complex relationship between glycolysis and OXPHOS [308]. The OXPHOS inhibitor elesclomol in combination with paclitaxel was clinically tested in a randomized trial in unselected patients. The study was terminated due to increased death in patients with high LDH receiving paclitaxel alone [309]. However, the study revealed a statistically significant increase in PFS in patients with normal serum levels of LDH receiving elesclomol, that is, a group of patients with tumors that rely on OXPHOS.

Regarding the possible effects of metabolic preferences of melanoma tumors on the efficacy of targeted therapy, it is of interest that mutant BRAF inhibition might be more efficacious in tumors with higher glycolytic index [170, 171]. As mentioned above, this metabolic trait could be useful as a predictive marker of response to BRAF inhibition. In addition, inhibition of BRAF(V600E) appears to induce a switch from glycolytic to OXPHOS metabolism, via induction of MITF, followed by MITF induction of PGC1α [172]. A consequence of this shift is an apparent dependency of BRAF-inhibited cells upon OXPHOS, thus suggesting new BRAF-combination strategies for patients. The role of glycolysis in responses of melanoma to mutant BRAF inhibition were highlighted in a study that demonstrated that concurrent inhibition of BRAF and glycolysis induces cell death in BRAF inhibitor-resistant melanoma cells [310].

The importance of OXPHOS in melanoma resistance to chemotherapy and targeted therapy with vemurafenib was highlighted in another study that aimed to explore the significance of a subpopulation of melanoma cells expressing H3K4 demethylase JARID1B. JARID1B melanoma cells cycle slowly, but can produce progeny of fast-dividing cells without having other hallmarks of stem cells [311]. Deeper analysis of these cells revealed that JARID1B positivity is associated with the post-treatment surviving fraction, and is marked by increased levels of OXPHOS which presumably contributes to their survival [312]. These resistant cells could be successfully targeted by inhibitors of OXPHOS such as phenformin, similar to findings in the study described above on MITF induction of PGCα. Phenformin, no longer used as antidiabetic, might still hold promise as a drug to combine with BRAF inhibitors as shown in a recent study [153], where it selectively eliminated the JARID1B positive cells left untouched by BRAF inhibition.

A most interesting study implicated enzyme PDH (pyruvate dehydrogenase) that links glycolysis to OXPHOS in the abrogation of mutant-BRAF-induced senescence (OIS). Oxygen consumption was increased in senescent cells modified to express mutant BRAF, which was mediated by activated PDH. Two key PDH-regulating enzymes were involved in the observed activation of PDH: inhibitory PDK1 was suppressed, while activating PDP2 was upregulated. Ectopic expression of PDK1 was able to overcome BRAF-induced senescence of melanocytes and promote robust tumor growth *in vivo*. Moreover, PDK1 depletion in melanoma tumors dramatically increased their sensitivity to vemurafenib *in vivo* by eliminating subpopulations of cells resistant to the drug [313]. These results established a direct role for metabolic axis in the development and drug resistance of melanomas.

The role of autophagy in tumor development and responses to various treatments remains controversial, most likely due to the fact that autophagy can contribute either to death or survival of cancer cells depending on the nature of the death signal and cellular context. A key autophagy protein ATG5 is reportedly downregulated in primary melanoma compared to nevi, and early stage melanomas with low ATG5 levels have worse prognosis [314]. This would suggest that autophagy in early stages might be limiting disease progression. Deprivation of amino acids is considered to be of potential benefit in melanoma treatment due to specific metabolic preferences/dependency of melanoma tumors, but induction of “starvation-induced” autophagy could be detrimental.

### Arginine deprivation

Melanoma tumors, along with hepatocellular cancer (HCC) and prostate cancer, frequently show deficiency of the enzyme argininosuccinate synthetase (ASS) [315], cannot synthesize arginine from citrulline, and depend on exogenous arginine for survival. Arginine degradation using arginine deiminase (ADI) leads to growth inhibition and eventually cell death, while normal cells that express ASS can survive (reviewed by Yoon[316]). Pegylated ADI (ADI-PEG20) has shown antitumor activity in melanoma [317]. Another arginine- degrading enzyme, arginase, in a recombinant-pegylated and cobalt-substituted form, Co-ArgI-PEG, is being developed for clinical trials. It is already clear that treatment with ADI-PEG20 induces resistance in patients by at least two identified mechanisms: induction of protective autophagy and re-expression of ASS and activation of the MAPK pathway (reviewed by Yoon et al. [316]). If arginine-deprivation therapy with arginine-degrading enzymes is to become a valid therapeutic option, concurrent therapies targeting autophagy or the MAPK pathway should be considered.

### Leucine deprivation

Leucine deprivation was also shown to induce apoptotic death in melanoma cells; moreover, the dependence of melanoma cells on leucine was functionally linked to the activated RAS-MAPK pathway, in particular BRAFV600E mutation. The latter rendered the mTOR pathway resistant to leucine deprivation, but inhibition of autophagy was able to restore sensitivity of these cells to leucine deprivation. Dietary leucine deprivation combined with an inhibitor of autophagy suppressed melanoma xenograft growth *in vivo* [318].

Autophagy is a potential target in melanoma. However, drug-induced autophagy could be either suicidal or protective, which is reflected in the ongoing clinical studies. Metformin was shown to induce suicidal autophagy in melanoma cells *in vitro* and *in vivo* [319]. Metformin, an inhibitor of mitochondrial complex I, activates AMPK as a result of decreased AMP/ATP ratio, and inhibits mTOR. Long known for its antihyperglycemic properties, it is being explored for antineoplastic activities. A trial of metformin with vemurafenib is ongoing (NCT01638676). In an opposing approach, the old antimalarial drug hydroxychloroquine, an inhibitor of autophagy, is also trialed with vemurafenib (NCT01897116).

Because autophagy is known to be involved in both innate and adaptive immunity, there is an interest in exploring manipulation of autophagy for immunotherapy. Autophagy was reported to enhance antigen presentation by dendritic cells, therefore potentiating T-cell activation, and could be enhanced by nanoparticle-based antigen presentation [320]; autophagosome preparations enriched for tumor antigens were used to pulse dendritic cells for successful vaccination of tumor-bearing mice [321]. On the other hand, inhibition of autophagy was shown to promote antitumor responses to systemic IL-2 immunotherapy [322, 323]. Apparently, the specific roles of autophagy in different immune responses would have to be explored individually.

### NEW PROGNOSTIC MARKERS

### Circulating melanoma cells

Detection of circulating tumor cells (CTCs) in melanoma was explored for at least last 20 years. CTCs have been shown to serve as seeds for metastatic lesions, and therefore their presence could be interpreted as a prognostic marker. In addition, monitoring of CTC levels could serve as a marker of disease progression and treatment success or failure (reviewed by Ireland et al. and Tanaka et al.[324, 325]). Molecular profiling of CTC could become a “liquid biopsy”, and could reflect genetic heterogeneity, both intratumoral or between multiple metastatic tumors.

The earlier attempts were directed mostly toward developing a multimarker RT-PCR to detect and monitor the presence of CTCs in the bloodstream. The first detection of melanoma CTCs was based on RT-PCR of tyrosinase [326]. Numerous studies have employed RT-PCR of a variety of melanoma markers to analyze peripheral blood preparations and examine prognostic significance of these assays. For example, a serial analysis of two melanoma-associated markers (tyrosinase and MART-1) was shown to be an independent predictor of disease recurrence for stage IV melanoma patients after surgery and adjuvant therapy [327]. A study that performed serial analysis of CTCs using a five-marker RT-PCR (MART-1, GalNAc-T, PAX-3, MITF and MAGE-A3) concluded that it could be useful for prognostic purposes [328]. CTC assessment with three markers—MART-1, MAGE-A3, and PAX3—provided prognostic discrimination before and during adjuvant treatment for resected stage IV melanoma patients [329]. Similarly, a stage III clinical trial found that positivity for at least two out of three CTC biomarkers (MART-1, GalNAc-T and MAGE-A3) examined was significantly associated with decreased recurrence-free survival in patients with stage II melanoma and metastases to sentinel lymph nodes [330]. These CTC markers were deemed to be useful in selecting patients for aggressive adjuvant treatments. A different set of CTC markers (MLANA, ABCB5, TGFbeta2, PAX3d and MCAM) was examined, and two of them (MLANA and ABCB5) were also found to be useful in predicting disease status [331]. At the same time, analysis of cytokine- receptor expression in CTCs had no predictive significance for treatment outcomes [332], possibly due to the choice markers. Overall, the many studies performed indicate that a validated set of CTC biomarkers as well as standardized methodology is necessary to make RT-PCR analysis of CTCs a useful tool in assessment of melanoma prognosis. For now, RT-PCR of peripheral blood is not considered to be highly promising.

A different approach involves physical separation of CTCs prior to their analyses. This methodology is somewhat limited by its most often used approach, which relies on expression of certain surface proteins on CTCs for their identification and separation. CellSearch Veridex technology, widely used for the detection of CTCs in blood, was designed for breast, prostate, colorectal, and lung cancer, which express EpCAM markers. Continuing expression of these proteins on CTCs could not be counted upon when de-differentiation (such as epithelial to mesenchyme transition in epithelial cancers) occurs during tumor progression. Nevertheless, use of the CellSearch platform was reported to detect CTCs in 40% of patients with advanced melanoma, and the number of CTCs was prognostic itself for overall survival and reflective of treatment outcomes [333]. The CellSearch Veridex platform was recently adapted for melanoma markers, and was reported to be successful in detection and quantification of CTCs in cerebrospinal fluid in two melanoma patients with leptomeningial metastases [334]

New techniques are being developed, such as isolation by size of epithelial tumor cells (ISET), which was able to detect CTCs in 29% of patients with primary invasive melanoma and in 62.5% of metastatic melanoma patients, with an excellent correlation for detection of CTCs by RT-PCR of tyrosinase [335]. Results obtained with ISET should, however, be considered carefully, because circulating melanocytes were detected in the blood of a patient with untypical melanocytic lesion [336]. A new technique named photoacoustic blood cancer testing was successfully applied to melanoma CTCs in mice and spiked human blood and could be used to capture CTCs [337, 338]. A recent report described a method to detect and isolate single circulating melanoma cells that integrates a polymer-nanofiber-embedded nanovelcro cell-affinity assay with a laser microdissection (LMD) technique. This method not only separates melanoma CTCs from peripheral blood, but also allows sequencing of individual cells for specific mutations [339].

The inertial focusing–enhanced microfluidic CTC capture platform, termed “CTC-iChip” is capable of rapid sorting of rare CTCs from whole blood. The iChip technology is capable of isolating CTCs using strategies that involve recognition of extracellular epitopes, but could be used independently of tumor-specific membrane-protein recognition [340]. The methodology was successfully tested in epithelial cancer and melanoma, and enables RNA profiling of single cells. Isolation of CTC using the ScreenCell filtration technique with quantitative analysis of CTC telomeres by TeloView was described recently [341].

Molecular profiling of CTC might provide a very valuable representation of a tumor(s) genotype because CTCs presumably reflect tumor heterogeneity better than a single tumor biopsy. Indeed, several studies have discovered discordant mutations in CTCs versus biopsies. Inconsistencies of BRAF and KIT mutations between tumor biopsies and CTCs were described [342, 343]. Presumably, CTCs also represent tumor cells with higher metastatic potential, and thus could be more relevant to the molecular profiling of aggressive tumor variants or metastases.

Several clinical trials are ongoing that incorporate CTC detection and analyses prior to and after treatments to investigate the prognostic value of CTCs. The CellSearch Veridex technique will be evaluated in terms of its predictive and prognostic ability (NCT01573494). CellSearch and Epispot (another platform based on immunomagnetic separation) are compared in NCT01558349. Trial NCT01528774 addresses the possibility of long-term culture of melanoma CTCs isolated using TrueCells technology. A future trial NCT01776905 will evaluate use of photoacoustic flow cytometry for *in vivo*, real-time detection of CTCs. Treatment with ipilimumab and stereotactic ablative radiation therapy (NCT01565837) or with Sargamostim/GM-CSF will include analysis of CTCs as predictive/prognostic markers (NCT01489423). Multiple parameters including CTCs are evaluated in an ongoing trial of imatininb (NCT00470470).

Recent studies have addressed the utility of yet another method of “liquid biopsy”, i.e., analysis of circulating tumor DNA (ctDNA) by NGS techniques rather than RT-PCR of defined biomarkers. A side-by-side comparison was performed on the analysis for a serum biomarker versus analysis of CTCs versus analysis of ctDNA by targeted- or whole-genomic sequence in breast cancer patients. This study reported the superiority of ctDNA analysis, as having a greater dynamic range, a greater correlation with changes in tumor burden, and better measure of treatment response [344]. The next publication from the same group reported monitoring of ctDNA by exomic sequencing in breast cancer patients prior to and during treatments [345]. Serial analysis of ctDNA had successfully detected a number of prognostic genomic alterations that were either increased or appeared *de novo* as a consequence of treatments. It remains to be seen if detection of ctDNA will be a useful prognostic companion or even a substitute for tumor biopsies.

### Prognostic test for uveal melanoma

Uveal melanoma is a distinct type of melanoma with a clinical course and molecular landscape of its own. Even though patients rarely present with metastatic disease and are routinely subjected to surgery with curative intent, almost half have a high-risk disease that requires careful monitoring and/or adjuvant therapy after tumor resection. It has been difficult to stratify patients into high versus low risk of metastatic disease based on clinicopathological findings only.

DecisionDx-UM gene expression profile test was developed as a stand-alone platform, which requires no other information for prognostic accuracy. The test is PCR-based and measures the expression of 12 selected genes from primary uveal melanoma samples obtained by fine needle biopsy. The test allows patients to be stratified into risk categories such that high-risk patients can be offered intensive metastatic surveillance and adjuvant therapy while low-risk patients can be spared unnecessary chemotherapy and frequent monitoring [346].

### Exosomes

Exosomes are small membrane vesicles with an endosome origin that are released by cells into the extracellular environment. They could fuse with other cells, thereby transferring the RNAs and proteins they carry. Tumor-derived exosomes are emerging mediators of tumorigenesis; there is a growing interest in exploring treatment options targeting exosomes. Exosomes are thought to participate in the formation of a pre-metastatic niche, i.e., a specific microenvironment that is reprogrammed to support survival and growth of metastatic cells. There has been significant interest in exploring the role of exosomes as prognostic markers (reviewed by Ohno [347]). A recent exciting study analyzed exosomes from highly metastatic melanomas. It found that these “metastatic” exosomes increased the invasive behavior of primary tumors by permanently “educating” bone marrow progenitors through the receptor tyrosine kinase MET. Melanoma-derived exosomes also induced vascular leakiness at premetastatic sites and reprogrammed bone marrow progenitors toward a pro-vasculogenic phenotype. Reducing MET expression in exosomes diminished the prometastatic behavior of bone marrow cells. Notably, MET expression was elevated in circulating bone marrow progenitors from individuals with metastatic melanoma. The study identified an exosome-specific melanoma signature with prognostic and therapeutic potential comprised of TYRP2, VLA-4, HSP70 (HSP90) isoform, and the MET oncoprotein [348].

## CONCLUDING REMARKS

It is very likely, considering the most recent findings, that the majority of driver mutations in melanoma have been identified. The study most quoted in this paper [32] reported that, outside of the most frequent mutations in BRAF and NRAS, driver mutations were identified in 83% of remaining BRAF and NRAS wild-type tumors. These results will be confirmed in much larger cohorts of melanoma tumors; some changes (identification of new rare-driver or helper mutations) could be anticipated. Indeed, new mutations are being discovered, such as recent identification of BRAF fusions in 4-8% of melanomas without mutations in the known “drivers”. However, while many genetic alterations in melanoma have been identified, the issue of tumor heterogeneity, within different tumors in the same patient, or even within individual tumor, could be expected to be a big problem both in melanoma biology and clinical approaches.

However, the accumulated knowledge is already sufficient to concentrate on the biological significance of genetic aberrations identified in melanoma and to evaluate their suitability as targets. Combination therapies are obviously the way of the future and might include two or more targeted therapies in combination with immunotherapies. One can also envision combinations that include novel therapies that target basic processes (e.g., metabolism and autophagy) that are sufficiently deranged in melanoma compared to normal cells.

The ubiquitous problem of resistance to targeted therapies could only be addressed by dedicated profiling of both inherent and acquired molecular features associated with resistance. This is a valid approach to either forestalling or overcoming resistance to targeted inhibitors, which is the biggest obstacle to development of meaningful precision treatment approaches in melanoma and other cancers.

The great promise of immune therapy in melanoma treatment is unlikely to be limited to the blockade of immune checkpoints. Combination of different approaches to activation of cytotoxic effector cells and inhibition of negative immune modulation is likely to have a higher rate of success than a single modality, as exemplified by combination of two immune checkpoint blocking antibodies. Biological basis of the sporadic nature of responses to long-approved immune treatments IL-2 and Ipilimumab are beginning to be understood, and hopefully will eventually translate into combination treatment that will increase the frequency of clinical responses.
